# Neurophysiological Basis of Emotional Face Perception and Working Memory Load in a Dual‐Task MEG Study

**DOI:** 10.1002/hbm.70242

**Published:** 2025-06-09

**Authors:** Katharina Lingelbach, Jochem W. Rieger

**Affiliations:** ^1^ Applied Neurocognitive Systems, Fraunhofer Institute for Industrial Engineering IAO Stuttgart Germany; ^2^ Applied Neurocognitive Psychology, Department of Psychology Carl von Ossietzky University Oldenburg Germany

**Keywords:** dual‐task, emotional face processing, event‐related magnetic fields (ERFs), eye‐tracking, magnetoencephalography (MEG), oscillations, working memory load

## Abstract

Research on the neurophysiological effects of emotional face processing, working memory (WM) load, and their interaction in dual‐tasks remains scarce. Therefore, we conducted a combined magnetoencephalography eye‐tracking study with 47 participants. The dual‐task temporally interleaved a facial emotion discrimination task with a visuo‐spatial n‐back task. Source‐space cluster analyzes of event‐related magnetic fields (ERFs) and oscillations revealed significant main effects of emotional expression and WM load. During emotion discrimination, enhanced ERFs for negative facial expressions located across the insula, ACC, and face‐specific occipital regions suggest amplified emotion processing but also the recruitment of attentional control mechanisms. During the n‐back phase, emotional faces did not affect evoked responses when they were task‐irrelevant. Interaction trends in pupil dilation indicated that emotion‐specific processing is diminished under high WM load. During the n‐back phase, increased WM load reduced alpha and low beta oscillations in temporo‐ and parieto‐occipital areas. In addition, reduced target fixations in the presence of negative facial distractors indicated a tendency toward emotion‐specific interference. Furthermore, sustained increased WM load affected perceived valence, pupil size, and reaction time in both subtasks. A convergence of neurophysiological, physiological, and behavioural findings points to specific processing modes with greater resource depletion for negative expressions and high WM load in the dual‐task. In conclusion, the study advanced our understanding of (a) circumstances under which emotional faces modulate ERFs in a dual‐task, (b) mechanisms underlying emotion discrimination, (c) interaction effects of emotional expression and WM load in gaze behavior, as well as (d) how WM‐related oscillatory alpha and beta power is affected by increasing load.

## Graphical abstract

We investigated emotional face processing and working memory in a dual‐task MEG‐eyetracking study. Task‐relevant negative faces increased evoked magnetic fields in regions associated with emotional face processing and attentional control, while high working memory load decreased alpha and beta power in temporo‐ and parieto‐occipital areas, suggesting greater resource depletion under these conditions. Gaze behavior was affected by an interaction between working memory load and emotional expression.

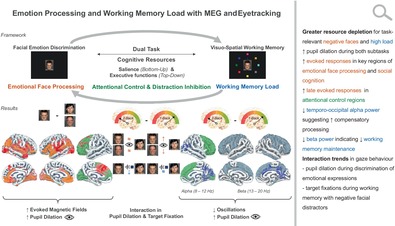

**Summary**

- Emotionally negative faces enhance evoked brain responses linked to face processing when task‐relevant, but not when acting as distractors.
- Working memory (WM) load modulates oscillatory signatures only during the encoding and retrieval phase. Decreases in parietal low beta band power suggest diminished WM maintenance under high load. In contrast, a decline in temporo‐occipital alpha power indicates enhanced attention allocation and information processing.
- Neurophysiological and behavioral findings suggest greater resource depletion and reduced attentional control for negative facial expressions and high WM load.
- Pupil dilation during emotion discrimination and target fixation during the WM encoding/retrieval phase indicate an interaction between load and emotion.

## 1 Introduction

In the past, cognitive and affective‐emotional processes have mostly been studied separately, ignoring their inherent interdependence in human nature (Cromheeke and Mueller 2014; Pessoa 2008). In the last decade, this perspective has shifted toward the study of functional brain networks and mechanisms underlying the interaction of emotion and cognition (Brockhoff et al. 2022; Schweizer et al. 2019).

One assumption of this research is that cognitive resources, including attentional control and the capacity to process and retain information, are limited (Baddeley 1992; Cowan 2017; Wickens 2014; Fougnie and Marois 2006). Attentional control facilitates goal‐directed behavior by guiding perception, processing, and response selection (Mackie et al. 2013; Miller and Cohen 2001), while working memory (WM) encodes, maintains, and retrieves task‐relevant information (Baddeley 1992, 1996; Eskikurt et al. 2024; Oberauer 2019). In dual‐tasks incorporating WM, a secondary task (e.g., discrimination task) and task‐irrelevant stimuli (e.g., a negative face) can divert attentional and processing resources from the primary task (e.g., maintaining multiple items in WM). This causes the risk of task interference and decay of stored information, especially as subtask demands increase (Cowan 2005, 2017; Navon and Miller 2002; Tombu and Jolicoeur 2003; Watanabe and Funahashi 2014).

### 1.1 Emotional Face Processing and Categorization in a Dual‐Task

Certain stimuli, such as faces, appear to be prioritized in processing regardless of task relevance (Lavie et al. 2004; Lavie and de Fockert 2005, but cf. Pessoa et al. 2002, 2005; Wang et al. 2016). Emotionally negative faces have been proposed to directly and automatically attract attention—even independently of available resources—due to their relevance for adaptive behavior (Adolphs 2003; Dolan 2002; Eimer and Holmes 2007; Roesch et al. 2010; Schindler and Bublatzky 2020; Schupp et al. 2006; Vuilleumier 2005). This bottom‐up phenomenon, referred to as emotional attention (Vuilleumier 2005; Vuilleumier and Huang 2009), enhances an early event‐related neurophysiological component named P1/P100 (Eimer and Holmes 2007; Schupp et al. 2006, but cf. Schindler and Bublatzky 2020).

**FIGURE 1:**
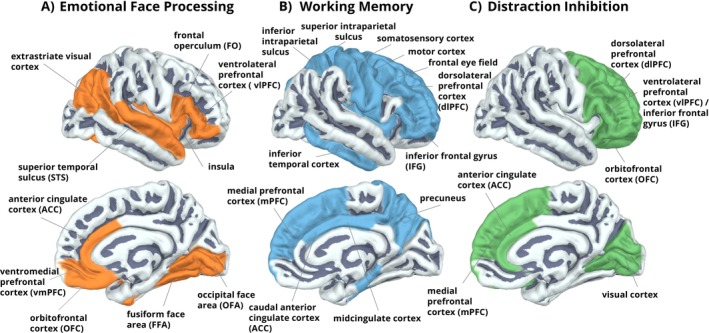
Functional brain networks involved in a dual‐task alternating between facial emotion discrimination and visuo‐spatial working memory load. Key regions from which activity can be measured with magnetoencephalography (MEG) are colored in orange, blue, and green for the subprocesses of (A) emotional face processing, (B) working memory, and (C) distraction inhibition.

However, further evidence suggests that emotions in expressions are processed more robustly at a later configural stage (see Schindler and Bublatzky 2020, for review; Hinojosa et al. 2015; Skinner and Benton 2010) and compete for cognitive resources during ongoing tasks (Hinojosa et al. 2015; Schindler and Bublatzky 2020). At this configural stage, emotional compared to neutral faces amplify a component emerging approximately 170 ms after face onset (N/M170; Schindler and Bublatzky 2020). The M170 is localized in the fusiform face area (FFA; Deffke et al. 2007; Halgren et al. 2000; Kanwisher et al. 1997; Monroe et al. 2013; Pizzagalli et al. 2002; see Figure 1A). Its deflection is more pronounced in the right hemisphere (Bentin and Deouell 2000) and correlates with reaction times in emotion discrimination tasks (Monroe et al. 2013).

The theory of prototype‐referenced shape encoding was proposed to explain mechanisms underlying the processing of configural facial features (Leopold et al. 2001, 2006). In contrast to bottom‐up mechanisms (Vuilleumier 2005; Vuilleumier and Huang 2009), this theory suggests a top‐down guided contrastive mechanism that compares figural variations with an internal face template. Deviations from this template enable the discrimination of facial configurations, including facial expression (Skinner and Benton 2010). Hence, the difficulty of discriminating a face increases with its resemblance to the internal template (top‐down effect). In cases of high similarity, amplified processing and greater attention toward the face may contribute to more accurate differentiation.

Figure 1A illustrates key brain areas involved in emotional face processing (Vuilleumier et al. 2003; Vuilleumier and Pourtois 2007; Winston et al. 2003; Posamentier and Abdi 2003; Weidner et al. 2024). Some of these regions are linked to the mirror neuron system and facilitate the interpretation of expressions (Carr et al. 2003; Haxby et al. 2000; Montgomery and Haxby 2008; Pitcher et al. 2008; Said et al. 2011).

Studies have demonstrated that increased perceptual or WM load can attenuate event‐related components associated with emotional face processing (Cao et al. 2022; van Dillen and Derks 2012; Wang et al. 2016; Yang et al. 2015, but cf. Müller‐Bardorff et al. 2018; Gläscher et al. 2007; see Brockhoff et al. 2022 for review), as well as the ability to discriminate facial expressions (Cao et al. 2022; Lim et al. 2014). Moreover, van Dillen and Koole (2009) reported a behavioral interaction between facial emotion and WM load, with longer reaction times for angry compared to happy faces only during low but not high load (see also van Dillen and Derks 2012). The authors attributed this phenomenon to increased attentional demands caused by the negative information. This interference effect is mitigated during high load due to increased attentional control toward the WM task to maintain task performance.

MacNamara et al. (2012) appears to be the only study to examine neurophysiological evidence alongside gaze behavior. Their study employed fearful and neutral facial distractors during a WM task with two load levels. The authors observed more fixations for neutral compared to fearful faces and high compared to low WM load, but no interaction effect between WM load and emotional distraction on gaze‐related measures. The faces were always presented as distractors during the WM maintenance phase. This leaves the question open of how WM load and emotionally negative and positive expressions affect gaze behavior in scenarios where faces are (1) context‐specific distractors or targets, and (2) presented not only during maintenance but also simultaneously during WM encoding and retrieval. Fixation duration and pupil size seem to reliably increase with emotional arousal (see Skaramagkas et al. 2021 for review), and provide useful measures for investigating interference as indicated by increased fixation duration and pupil size for negative facial distractors and targets across WM phases. Unfortunately, MacNamara et al. (2012) did not include these gaze measures in their analysis.

### 1.2 WM Load in a Dual‐Task

WM requires an interplay of attention, perception, and response selection (see Figure 1B; for meta‐analyses, see D'Esposito and Postle 2015; Kim 2019; Rottschy et al. 2012).

Neuroimaging studies reported increased activation in the anterior insula, medial prefrontal cortex (mPFC) and inferior frontal gyrus (IFG) with increasing load (Cairo et al. 2004; Rottschy et al. 2012; Tomasi et al. 2007; see Figure 1B). However, during high WM load, where capacity reaches its limits, neural activation can be attenuated in the primary motor and sensory cortices, precuneus, and anterior temporal lobe (Chen et al. 2023; Michels et al. 2010; see Figure 1B). Such reduced activation during high compared to low WM load appears to be correlated with performance and an attenuated representation of task‐relevant information (Leung et al. 2004; Michels et al. 2010). Previous magneto‐ and electrophysiological research suggests that WM load modulates oscillatory power in different frequency bands (for review, see Pavlov and Kotchoubey 2022): As WM load increases, increased frontal theta and decreased parietal alpha and beta power were found (Scharinger et al. 2015, 2017). However, the role and direction of oscillatory modulations in the alpha and beta band and their link to increasing visual WM load remain inconclusive (Chen and Huang 2015; Pavlov and Kotchoubey 2022; Proskovec, Wiesman, et al. 2019; Proskovec, Heinrichs‐Graham, et al. 2019). Notably, Pavlov and Kotchoubey (2022) reported that in over 80% of the reviewed studies, WM load modulated alpha and beta band oscillations in the same direction, either both increasing or decreasing. In their MEG study, Proskovec, Heinrichs‐Graham, et al. (2019) found that high WM load enhanced occipital alpha activity during maintenance but decreased it during encoding. Notably, high‐load WM simultaneously caused greater alpha suppression in parietal cortices during maintenance, contrasting with load‐related alpha power increases in occipital areas (see also Michels et al. 2010). The authors concluded that whether alpha band power increases or decreases with increasing load depends on the specific brain region (occipital vs. parietal) and WM phase (encoding vs. maintenance; Proskovec, Heinrichs‐Graham, et al. 2019). Alpha oscillations are assumed to act as a gating mechanism for top‐down allocation of attention to task‐relevant and suppression of task‐irrelevant neural activity (Bonnefond and Jensen 2012; Boonstra et al. 2013; Jokisch and Jensen 2007; Klimesch et al. 2007; Schroeder et al. 2018; Proskovec, Wiesman, et al. 2019; Fries et al. 2001). The neural suppression protects task‐relevant information and activity from interference (Chen and Huang 2015; Gevins et al. 1997; Pavlov and Kotchoubey 2022). In line with this assumption, temporo‐occipital alpha band modulations appear to correlate with individual performance differences (Bonnefond and Jensen 2012; Proskovec, Wiesman, et al. 2019).

At the behavioral level, WM retrieval accuracy declines and reaction time increases as WM load increases (van Dillen and Derks 2012; Yang et al. 2015; Wang et al. 2016; MacNamara et al. 2012; Tavares et al. 2016). Increasing load also affects gaze behavior, leading to increased fixation durations and a larger pupil size (Skaramagkas et al. 2021).

#### 1.2.1 Emotional Distractor Inhibition During WM

Several studies have shown that prefrontal areas play a role in goal‐directed attention control and resource allocation to suppress effects of distracting task‐irrelevant emotional information (Dolcos and Denkova 2014; García‐Pacios et al. 2015b, 2017; Iordan et al. 2013; Ochsner and Gross 2005; Yoon et al. 2006). Compared to neutral distractors, emotional distractors appear to attenuate and even disrupt WM‐related neural processes (see Schweizer et al. 2019 for a meta‐analysis and Dolcos and Denkova 2014; Dolcos et al. 2011 for reviews). Regulatory frontal areas that counteract these distraction effects comprise dorsal areas including the dorsolateral PFC (dlPFC), mPFC, ACC, as well as ventral regions including the ventrolateral PFC (vlPFC), OFC, and IFG (Ochsner and Gross 2005; see Figure 1C).

Studies investigating oscillatory processes reported an increase in posterior alpha power in response to visual distractions, suggesting enhanced top‐down suppression of irrelevant neural activity (Bonnefond and Jensen 2012; Boonstra et al. 2013; Jokisch and Jensen 2007; Klimesch et al. 2007; Schroeder et al. 2018).

A magnetoencephalography (MEG) study has yielded insights into the temporal dynamics of emotional image distraction inhibition in a delayed‐recognition paradigm (García‐Pacios et al. 2015b, 2017). These temporal dynamics revealed that functional connectivity of frontal and parietal regions was reduced in early time windows due to the detection of emotional distractors (50–150 ms after distractor onset). This was followed by the involvement of prefrontal control processes to suppress distractions and re‐establish the fronto‐parietal WM processes (250–460 ms after distractor onset; García‐Pacios et al. 2015b, 2017). The pattern was particularly pronounced for emotionally negative distractions (García‐Pacios et al. 2015b, 2017). Behaviorally, García‐Pacios et al. (2015a) reported increased reaction times and reduced accuracy during negative compared to positive and neutral distraction, whereas no differences were observed between positive and neutral images. As one of the few studies assessing subjectively perceived valence and arousal of distractor images, they found that participants rated positive images highest in valence, followed by neutral and negative images. In contrast, arousal ratings were highest for negative images, followed by positive and neutral images (García‐Pacios et al. 2015a). Other behavioral studies have reported interaction effects between WM load and the valence of distractors. WM performance declined during high but not low load scenarios for negative compared to positive and neutral distractors (Tavares et al. 2016; Li et al. 2012 for image distractors; cf. Schweizer et al. 2019 for a meta‐analysis; and cf. van Dillen and Derks 2012; Yang et al. 2015; Wang et al. 2016; MacNamara et al. 2012 for facial distractors).

### 1.3 Research Question

Taken together, existing evidence on interaction effects between WM load and emotional expressions primarily stems from electrophysiological (Brockhoff et al. 2022; van Dillen and Derks 2012; Schindler and Bublatzky 2020) or neuroimaging studies (Gläscher et al. 2007; Müller‐Bardorff et al. 2018), often without the inclusion of gaze behavior (but see MacNamara et al. 2012) and with either poor spatial or temporal resolution. Most of the studies did not examine interaction effects on a face‐related task (but see van Dillen and Derks 2012; Van Dillen and Koole 2009) and none used a task explicitly emphasizing the emotional expressions. Some studies employed only one WM load level (García‐Pacios et al. 2015b, 2017). Others were limited to two emotion conditions (negative vs. positive in van Dillen and Derks 2012; negative vs. neutral in MacNamara et al. 2012; Tavares et al. 2016) or used images as emotional stimuli (García‐Pacios et al. 2017; Tavares et al. 2016; Li et al. 2012). In addition, all existing studies focused on distraction effects during WM maintenance (García‐Pacios et al. 2017; Li et al. 2012; van Dillen and Derks 2012; Yang et al. 2015; MacNamara et al. 2012; Tavares et al. 2016) without investigating effects during WM encoding/retrieval. Finally, electrophysiological studies on emotional face processing under different load levels have reported inconsistent findings regarding which components and processes are modulated by an interaction (Brockhoff et al. 2022; van Dillen and Derks 2012; Müller‐Bardorff et al. 2018; Schindler and Bublatzky 2020; Yang et al. 2015).

**FIGURE 2:**
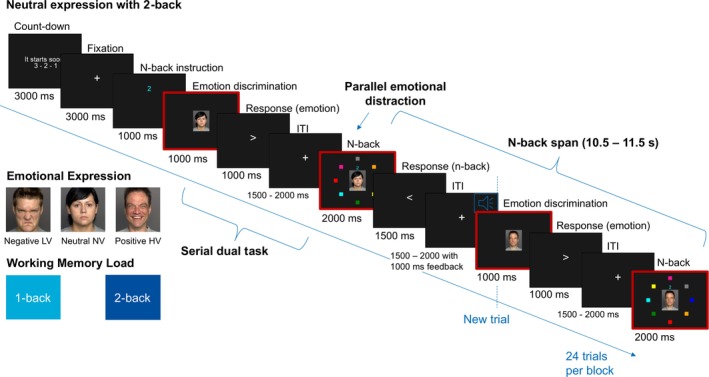
Dual‐task experiment alternating between facial emotion discrimination with three emotional expressions (LV: low valence, NV: neutral valence, and HV: high valence) and a color‐based spatial n‐back subtask with two working memory (WM) load levels (1‐ and 2‐back). ITI: Inter‐stimulus interval. WM maintenance is required during the emotion discrimination phase. WM encoding of the new target position and retrieval (i.e., matching of the target position with n trials back) co‐occur during the n‐back phase. Across a block, emotional expression and WM load level remain the same. Facial stimuli are task‐relevant in the emotion discrimination phase, while they are task‐irrelevant in the n‐back phase.

In sum, the evidence for neurophysiological, gaze‐related, and behavioral effects of multiple WM load levels and phases, as well as facial distractors including negative, neutral, and positive expressions, remains inconclusive. To address this, the present study examines the effects of WM load and emotion, and specifically their interaction, on (a) neurophysiological dynamics, (b) oscillatory signatures, (c) gaze behavior, (d) performance, and (e) subjective ratings during facial emotion discrimination and WM in a dual task. For the WM load manipulation, we employed an n‐back paradigm with two load levels: 1‐back (low WM load; LW) and 2‐back (high WM load; HW). The n‐back task is widely used to investigate WM (Kim 2019; Schmiedek et al. 2014) and is well‐suited for dual‐task paradigms (e.g., Kimura and Matsuura 2023; Unni et al. 2017). Our experimental paradigm required participants to maintain n‐back‐related information in WM while simultaneously performing the emotion discrimination subtask (i.e., facial emotion discrimination phase with WM maintenance). To assess facial emotion effects, happy (high valence; HV), neutral (average valence; NV), and angry (low valence; LV) expressions were used as target stimuli in the discrimination task. In the subsequent visuo‐spatial WM encoding/retrieval phase, the same faces appeared as distractors while participants compared and updated n‐back target information (see Figure 2). Brain activity and gaze behavior were simultaneously recorded using whole‐head MEG and eye‐tracking.

Given that previous research has only investigated isolated aspects of interacting emotion processing and WM load in the visual domain (regarding stimulus material, number of load levels, and time point of distraction), the current study aims to provide a more comprehensive understanding through the dual‐task paradigm. It integrates multimodal evidence to investigate the circumstances under which interaction effects occur and how they manifest in emotional face processing and visuo‐spatial WM.

#### 1.3.1 Hypotheses

Table 1 provides an overview of the hypotheses investigated in this study, structured by measure, dual‐task phase, and effect type. In subsequent sections, hypotheses are referenced by their respective IDs.

**TABLE 1:** Summary of the hypotheses, organized by measure (a–e), task phase (facial emotion discrimination with WM maintenance and visuo‐spatial WM with encoding/retrieval), and effect type (main and interaction effect).

H.ID	(a) MEG ERF	(b) MEG Oscillation	(c) Gaze Behavior	(d) Performance	(e) Ratings
Facial emotion discrimination phase					
Main effect of emotional expressions (LV–NV and HV–NV)					
1	Modulated HV^a^	x	↓ Fixation duration HV^b^, ^c^	↓ Errors HV^c^	↑ Valence HV^d^
	Modulated LV^a^		↑ Fixation duration LV^b^, ^c^	≃ Errors LV^c^	↓ Valence LV^d^
2	↑ Early processing (~P1) HV^a^		↓ Fixation count HV^b^, ^c^	↓ Reaction time HV^c^	↑ Arousal HV^d^
	↑ Early processing (~P1) LV^a^		↑ Fixation count LV^b^, ^c^	≃ Reaction time LV^c^	↑ Arousal LV^d^
3	↑ Configural processing (~M170) HV^a^		↓ Pupil dilation HV^b^		↓ Effort HV^h^
	↑ Configural processing (∼ M170) LV^a^		↑ Pupil dilation LV^b^		↑ Effort LV^h^
4	↑ Evaluative processing (∼ LPP) HV^a^				
	↑ evaluative processing (∼ LPP) LV^a^				
Dual‐task main effect of WM load during maintenance (HW–LW)					
5	x	Modulated^e^	↑ Fixation duration^b^	↑ Errors^e^	↓ Valence^h^
6		↑ Frontal θ ^e^	↑ Fixation count^b^	↑ Reaction time^e^	↑ Arousal^h^
7		↓ Parietal α/β ^e^	↑ Pupil dilation^b^		↑ Effort^e^
8		↑ Occipital α ^e^			
	Interaction Effect (LV–NV/HV–NV HW–LW) ⇒ ≃ NV in HW^i^		Interaction Effect (HV–NV HW–LW) ⇒ ≃ NV in HW		
**9**	x Modulated HV^a^, ^h^	Modulated^d^, ^e^, ^g^	↑ Fixation duration^f^, ^h^	↑ Errors^f^	↓ Valence^h^
	x Modulated LV^a^				
**10**	↓ Early processing (∼ P1) HV^a^, ^h^	↓ Frontal θ ^d^, ^e^	↑ Fixation count^f^, ^h^	↑ Reaction time^f^	↑ effort^h^
	↓ Early processing (∼ P1) LV^a^				
**11**	↓ Configural processing (∼ M170) HV^a^, ^h^	↑ Parietal α/β ^d^, ^e^	↑ pupil dilation^f^, ^h^		
	↓ Configural processing (∼ M170) LV^a^				
**12**	↓ Evaluative processing (∼ LPP) HV^a^, ^h^				
	↓ Evaluative processing (∼ LPP) LV^a^				
Visuo‐spatial WM encoding/retrieval phase					
Main effect of WM load during encoding/retrieval (HW–LW)					
13	x	Modulated^e^	↑ Target fixation^b^	↑ Errors^e^	↑ Effort^e^
14		↑ Frontal θ ^e^	↑ Target onset^b^	↑ Reaction time^e^	
15		↓ Parietal α/β ^e^	↑ Pupil dilation^b^		
16		↑ Occipital α ^e^	↓ Face fixation^h^		
	Depleted cognitive resources and attentional control ⇒ Interaction effect HW–LW only for LV^j^				
**17**	Modulated^d^, ^g^	Modulated^d^, ^g^	↓ Target fixation^g^	↑ Errors^g^	↑ Effort^g^
**18**	↑ Early processing (∼ P1)^a^, ^g^	↓ Frontal θ ^d^, ^e^, ^g^	↑ Face fixation^g^	↑ Reaction time^g^	
**19**	↑ Configural processing (∼ M170)^a^, ^g^	↑ Parietal α/β ^d^, ^e^, ^g^			
**20**	↑ Evaluative processing (∼ LPP)^a^, ^g^	↓ Occipital α ^d^, ^e^, ^g^			

*Note:* Main hypotheses are highlighted with blue‐colored, bold row numbers, and blue‐colored subheadings. Hypotheses highlighted in green are supported by our results.

Abbreviations: ERFs: event‐related fields; H.ID: hypothesis ID; HV: high valence; HW: high WM load; LPP: late positive potential; LV: low valence; LW: low WM load; NV: neutral valence.

^a^ Brockhoff et al. (2022), Schindler and Bublatzky (2020).

^b^ Skaramagkas et al. (2021).

^c^ Becker and Rheem (2020), Eastwood et al. (2003), and Weidner et al. (2024).

^d^ García‐Pacios et al. (2017).

^e^ Pavlov and Kotchoubey (2022), Michels et al. (2010), Bonnefond and Jensen (2012), Proskovec, Wiesman, et al. (2019), Proskovec, Heinrichs‐Graham, et al. (2019), Scharinger et al. (2015, 2017).

^f^ Van Dillen and Koole (2009) and van Dillen and Derks (2012).

^g^ Schweizer et al. (2019).

^h^ Hypotheses are marked as exploratory due to a lack of existing research.

^i^ Effects are assumed to be stronger for LV compared to HV (García‐Pacios et al. 2017; Van Dillen and Koole 2009; van Dillen and Derks 2012).

^j^ The combination of LV and HW is assumed to enhance gaze‐related, behavioral, and subjective WM load correlates without changing the effect's direction.

Based on previous research, we hypothesized that the processing of emotional facial expressions would modulate event‐related magnetic fields (ERFs; H.1–4a and H.17–20a), while WM load would manifest in changes of oscillations (during maintenance; H.5–8b; and encoding/retrieval; H.13–16b).

##### 1.3.1.1 Facial Emotion Discrimination Phase

During the discrimination, we expected a main effect of emotional expression on ERFs associated with emotional face processing (H.1–4a). Positive faces should lead to a processing advantage in gaze‐related measures (H.1–4c), performance (H.1–2d) and ratings (H.1–3e). Concurrent high WM load induced by the n‐back subtask is assumed to modulate oscillatory power in frequency bands linked to WM maintenance (H.5–8b) and affect gaze‐related measures (H.5–8c), performance (H.5–8d), and ratings (H.5–8e).

*Interaction*: An interaction effect with WM load is hypothesized to manifest in the form of attenuated emotion‐related ERF modulations (H.9–12a) and maintenance‐related oscillatory signatures (H.9–12b). This effect should be particularly visible for negative faces (García‐Pacios et al. 2015b, 2017; Schweizer et al. 2019). For positive faces, a high WM load should mitigate any processing advantages observed in gaze‐related measures and performance (H.9–11c–e).

##### 1.3.1.2 Visuo‐Spatial WM Encoding/Retrieval Phase

During the n‐back phase, we predicted a main effect of WM load on oscillatory signatures (H.13b), characterized by increased frontal theta power (H.14b) and decreased parietal alpha and beta power (H.15b). To regulate effects of task‐irrelevant facial distractors, posterior (occipital and temporal) alpha power should suppress irrelevant neural activity associated with emotional face processing, assuming cognitive resources for attentional control are available (H.13a,16b). Gaze‐related and behavioral measures should reflect goal‐directed attention allocation (H.13–14, 16c) and an increase in cognitive effort during high WM (H.13–16c–e).

*Interaction*: However, for negative facial expressions combined with a high WM load, we anticipated an interaction effect with task interference due to heightened processing demands and depleted resources (H.17–20a–e). Consequently, due to loss of attentional control, we expected to observe enhanced ERFs related to task‐irrelevant emotional face processing during the n‐back encoding/retrieval phase (H.17–20a,c), along with reduced WM‐related frontal theta (H.18b) and increased parietal alpha and beta power (H.19b), as well as decreased regulatory posterior alpha power (H.20b).

## 2 Methods and Materials

### 2.1 Participants

A total of 47 healthy volunteers (mean age = 24.87 years, SD = 3.28, range: 19–33 years, 27 females, and 20 males) participated in the study. A power analysis was conducted, indicating a required sample size of at least 36 participants to detect an effect of small to medium size (*η*_p_^2^ = 0.03) with an α of 0.05, power (1 − *β*) of 0.8, and estimated correlation of repeated measures at *r* = 0.5.

All participants were right‐handed, had normal or corrected‐to‐normal vision, and reported no color blindness, neurological diseases, psychiatric disorders, or consumption of psychoactive medication or drugs. They gave written informed consent before the experiment and received monetary compensation for their participation. The experiment was approved by the Commission for Research Impact Assessment and Ethics at the Carl von Ossietzky University, Oldenburg, Germany (Ref: EK/2018/070) and conducted in compliance with the Declaration of Helsinki.

For the MEG source‐space analyses, we excluded participants with technical issues (*n* = 5), missing anatomical T1 due to claustrophobic attacks in the MRI scanner (*n* = 3), and a very low n‐back performance of below 50% accuracy (*n* = 1). This exclusion resulted in 38 MEG datasets (mean age = 24.87 years, SD = 3.36, age range: 19–33 years, with 22 females and 16 males). For the eye‐tracking analysis, seven participants were excluded due to insufficient calibration or data quality (*N* = 40, mean age = 24.68 years, SD = 3.07, age range: 19–33 years, with 25 females and 15 males).

### 2.2 Procedure and Material

The experiment comprised 18 pseudo‐randomized blocks, divided into three rounds, with six blocks per round and 24 trials within each block (see Figure S1). Within each round, the six conditions (two WM levels × three emotional expressions) were presented in random order. Each block started with a countdown followed by a fixation cross presented for 3000 ms. Afterwards, participants had to perform a dual‐task with two subtasks in each trial. First, they were given a facial emotion discrimination task followed by a visuo‐spatial n‐back task (Figure 2). At the end of each block, participants rated their overall perceived effort using an adapted version of the NASA Task Load Index (NASA‐TLX) subscale (Hart and Staveland 1988) as well as the overall arousal and valence using the Self‐Assessment Manikin (SAM) subscales (Bradley and Lang 1994). Participants underwent two practice blocks at the beginning of the experiment to ensure sufficient task comprehension.

### 2.3 Facial Emotion Discrimination Phase

Face images used for emotion discrimination were obtained from the validated FACES database (Ebner et al. 2010) after permission was granted. The database comprises 513 naturalistic Caucasian faces. Each identity was depicted in three images with either a happy (high valence, HV or positive), angry (low valence, LV or negative), or neutral expression (neutral/average valence, NV; see Figure 2). Images were balanced for each condition regarding gender and age group (young, middle‐aged, and elderly faces). Within each trial, a face image was presented for 1000 ms in the middle of the screen and participants had to discriminate whether the face had a neutral or emotional expression. The facial expression condition (LV, NV, or HV) was constant throughout a block, but identities were randomly selected from the pool of 171 faces without repetition of the same image within the block.

### 2.4 Visuo‐Spatial N‐Back Phase

The color‐based visuo‐spatial n‐back task was an adapted version of von Lühmann et al. (2019). Eight colored squares (red, magenta, blue, turquoise, green, yellow, orange, and grey) were arranged equidistantly in a circle. In the middle of the circle, the static face image of the previous facial emotion discrimination phase was repeated to achieve a simultaneous emotion‐based interaction during the n‐back encoding/retrieval phase. Above the face image, a colored target number indicated the current target color and n‐back level (Figure 2). The colored target number remained the same over a whole block and was initially presented at the beginning of each block for 1000 ms (Figure 2; n‐back instruction in Figure S1). The color of each square changed trial‐wise within the block. For each trial, they had to compare whether the position of their target color was either the same as *n* trials before (same n‐back position) or not (different n‐back position). Participants performed two WM load levels—a low WM load condition with a 1‐back and a high one with a 2‐back. Since the n‐back interval of remembering the position between trials was quite long, at 10.5–11.5 s, we decided against choosing a high WM load condition above 2‐back.

### 2.5 Response Format

We used two response pads with the buttons positioned below the index fingers of the left and right hands. To avoid a fixed button‐to‐response mapping, resulting in anticipation effect during the face image and n‐back presentation, mapping changed on each trial and was indicated by the direction of an arrow (Figure 2). The arrow was presented for 1000 ms after the emotion discrimination phase and 1500 ms after the n‐back phase. It pointed to the button mapped to an emotional facial expression or the same n‐back position. In case of an emotional facial expression or the same n‐back position, participants had to press the button the arrow pointed to as quickly as possible. In the cases of a neutral face or different n‐back position, they had to press the button opposite the arrow point. The response interval was followed by an inter‐stimulus interval (ITI) with a jittered length of 1500–2000 ms. In the 1000 ms of the ITI after the n‐back encoding/retrieval phase, they received auditory feedback via in‐ear headphones on whether their n‐back response was correct or not. We did not provide feedback on emotion discrimination to avoid disrupting subsequent emotion‐based effects.

### 2.6 Data Acquisition

The experimental dual‐task was created and presented using Python 3.7 and PyGame (1.9.6). We used a parallel port and Expyriment (0.10.0) to send synchronization and response triggers to the MEG system. The dual‐task was displayed using rear projection on a screen measuring 750 × 428 mm inside the MEG chamber with a resolution of 1400 × 1050 pixels (Panasonic PT‐DS 12 KE, 60 Hz refresh rate). The back‐projection screen was positioned 115 cm away from the participant's eyes. The screen covered 15.07° of visual angle in width and 8.73° in height from the center. The face stimuli were centrally presented as rectangles on a dark grey background (#191919), each extending 3.30° of visual angle in height and 3.46° in width. The circle of squares had a radius of 7.60° (outer) and 6.41° (inner) of visual angle in height and 9.95° (outer) and 8.40° (inner) of visual angle in width from the center.

#### 2.6.1 Eye‐Tracking

We recorded gaze behavior during the experiment using a MEG‐compatible infrared eye‐tracking device (EyeLink 1000 Plus; SR Research Ltd., Ottawa, Canada) with a 1 kHz sampling rate. A 9‐point calibration was performed at the beginning of each experiment and a drift correction at the beginning of each block. The average calibration error was kept below 0.5° of visual angle (*max* < 1.0°).

#### 2.6.2 MEG

Neuromagnetic signals were recorded using a 306‐channel whole‐head MEG system (Elekta Neuromag Triux, Elekta Oy, Helsinki, Finland) with 102 magnetometer and 204 orthogonal planar gradiometer sensors. The MEG system was located inside a magnetically shielded chamber (Vacuumschmelze, Hanau, Germany). The dewar was positioned at 68° with participants seated underneath the MEG sensors in an upright position. Five head position indicator coils (HPI) were attached to the participant's head to enable continuous position tracking during the recording. We digitized coil positions along with anatomical landmarks (nasion, left; LPA; and right pre‐auricular; RPA; points) and at least 200 head‐shape samples (Whalen et al. 2008) using a Polhemus Fastrak (Polhemus, Colchester, VT, USA) to later co‐register the MEG data with the structural T1 MRI scans. The MEG signals were recorded without internal active shielding, at a sampling rate of 1 kHz, and with an analogue online bandpass filtering between 0.1 Hz and 330 Hz.

#### 2.6.3 Magnetic Resonance Imaging (MRI) Acquisition

To analyze the MEG data in source space, two structural T1‐weighted MRI scans were obtained from each participant, which were averaged to get a better signal‐to‐noise ratio (SNR). Images were acquired using a Siemens Magnetom Prisma 3.0 Tesla whole‐body MRI machine (Siemens, Erlangen, Germany) with a 3D T1‐weighted sequence (MPRAGE, TR = 2000 ms, TE = 2.07 ms, flip angle = 9°, voxel size = 0.75 × 0.75 × 0.75 mm^3^, GRAPPA = 2, field of view = 240 × 240, 224 sagittal slices, fat‐saturated, TA = 7:45 min). The T1 images were segmented into specific brain tissues (i.e., white matter, brain, scull, and skin) and individual brain surfaces were reconstructed for source localization using FreeSurfer (v. 6.0.0; Dale et al. 1999; Fischl et al. 1999).

### 2.7 MEG Analysis

The analyses were performed using Python 3.9 and MNE‐Python (v. 1.3; Gramfort et al. 2013).

#### 2.7.1 Preprocessing in Sensor Space

To mitigate the influence of external noise, we applied a Maxwell filter to the MEG data, as implemented in MNE‐Python (Taulu et al. 2005; Taulu and Simola 2006) with the default settings (*L*_in_ = 8, *L*_out_ = 3, correlation limit between inner and outer subspaces = 0.98; Taulu et al. 2005; Taulu and Simola 2006). The filter uses spatiotemporal signal space separation (tSSS) to decompose the neuromagnetic signal into spatiotemporal components originating from inside and outside the sensor helmet. Magnetic interference that does not originate from brain sources can thereby be attenuated. The Maxwell filter further allows for interpolating bad channels, transforming the data into a common coordinate system with equal origin for all rounds, and compensating for head movements by transforming the signals to the initial head position based on the continuous HPI coil tracking. For three subjects, the continuous HPI coil tracking could not be properly recorded due to a technical issue. In these cases, no head movement correction was applied. The head movement correction quality was overall high, with a goodness of fit for the head position indicator coils exceeding 0.99 on a scale of 0 to 1 across all participants. The average 3D head movement distance to the initial point, calculated from the indicator coils, was 3.04 mm (SD = 1.78 mm), and 47.09% of the distances were below 2 mm. Additionally, the average 3D head movement distance between consecutive samples (sampled at 1 kHz) was 0.18 mm (SD = 0.13 mm), with 97.85% of the distances falling within a 2 mm threshold. After applying the Maxwell filter, raw data were downsampled to 100 Hz (Fourier method with a boxcar window; as implemented in scipy v. 1.11.4) and band‐pass filtered using a 4th‐order infinite impulse response (IIR) Butterworth filter. In the time‐domain analyses, a narrow frequency band was chosen with cutoff frequencies set at 0.1 and 20 Hz. In the frequency domain analyses, the chosen cutoffs were 0.1 and 42 Hz.

We analyzed two time windows of interest: the time interval starting from the presentation of the face during emotion discrimination and from the presentation of the n‐back wheel with concurrent facial distractor during the visuo‐spatial WM encoding/retrieval (Figure 2; red highlighted frames).

The filtered signals were segmented into epochs, each lasting from 200 ms before stimulus onset to 1000 ms after the onset. For analysis of oscillatory power during the n‐back encoding/retrieval phase, a larger time interval was selected, extending from 200 ms before to 2000 ms after the stimulus onset. We concatenated epochs from the three rounds and exclusively selected the magnetometer data for further analyses. We focus our analysis on magnetometers because they allow us to probe deeper sources (Hämäläinen et al. 1993), such as orbitofrontal regions, the insula, or the cingulate cortex. These areas were revealed as key regions in prior studies investigating cognitive control mechanisms and emotional processing (Dong et al. 2024; García‐Pacios et al. 2017; Iordan et al. 2013).

To remove cardiac‐, blink‐, and muscle‐related artifacts, we performed a semi‐automated independent component analysis (ICA) on the epoched data using the FASTER pipeline (v. 1.2; Nolan et al. 2010) and MNE‐Python (Gramfort et al. 2013). We performed the ICA with components capturing at least 99% of the explained variance (*M* = 55.24, SD = 3.41 components for the time‐based analysis and *M* = 57.57, SD = 2.88 for the frequency‐based analysis) and using the extended infomax algorithm (Lee et al. 1999). Components contaminated with eye movements were manually selected after visual inspection of the topography, time course, and power spectrum (Chaumon et al. 2015; Hipp and Siegel 2013). Afterwards, the automated FASTER algorithm was applied to remove further contaminated components with features suggested by Nolan et al. (2010). Electrocardiac signals were reconstructed from the MEG sensors to remove cardiac‐related artifacts via the FASTER algorithm. For the time‐based analysis, an average of 6.62 components (SD = 1.17; min = 4, max = 9) were removed per participant, while for the frequency‐based analysis, an average of 7.26 components (SD = 1.38; min = 5, max = 11) were removed before back‐projecting the signals into sensor space.

In the last step, we performed an epoch‐wise baseline correction by subtracting the mean of the 200 ms window before the stimulus onset. We excluded the initial epoch from each 1‐back block and the first two epochs from each 2‐back block. This was necessary because the n‐back load had not fully developed during these trials, resulting in a lower WM load as intended in the respective condition.

#### 2.7.2 Source Space Transformation

We reconstructed and localized the sources of the neural activity from the MEG sensor data (Hämäläinen et al. 1993).

To model the conductivity in the brain, we chose the numerical method of a boundary‐element model (BEM; Mosher et al. 1999) with the FreeSurfer watershed tessellation algorithm (Ségonne et al. 2004). A single‐shell head model with the triangulation of the inner skull was used to compute the geometry information in the form of the BEM solution with a conductivity value of 0.3 Siemens/m.

The source space was created using a uniformly distributed grid of dipoles with positions and orientations according to the MNI305 (Montreal Neurologic Institute) space (Collins et al. 1994) as implemented in FreeSurfer. We used icosahedron subdivisions with distance‐based spacing of 5 mm yielding approximately 10,242 sources per hemisphere and a source spacing of 3.1 mm. This comprises a surface area per source of 9.8 mm^2^. A neuroanatomical label was automatically assigned to each grid point based on the Desikan‐Killiany atlas (Desikan et al. 2006; Fischl et al. 2004).

We performed the coregistration in a semi‐automated approach following Houck and Claus (2020). First, anatomical landmarks in the MRI headspace (i.e., the fiducial points: LPA, RPA, and nasion) were estimated from an MNI305 brain template (fsaverage; Dale et al. 1999; Gramfort et al. 2013) and transformed to each participant's MRI coordinate space. The estimated landmarks were visually inspected for each subject and manually adjusted to enhance accuracy. In the next step, an initial fit including scaling, translation, and rotation to the MRI head surface was performed using only the fiducial points (relative weight for nasion = 10, LPA and RPA = 1). Next, head shape points and MRI were automatically aligned using the iterative closest point (ICP) algorithm (6 iterations, nasion weight = 2, other points' weight = 1). In the third step, head shape points with a distance larger than 5 mm to the MRI skin surface were omitted. In the last step, the ICP algorithm was repeated with 20 iterations (nasion weight = 2). In two participants, the recorded nasion landmark was slightly distorted compared to the rest of the head shape points and fiducials. Therefore, we reduced the nasion weight parameter to 1 and increased the weight of the RPA and LPA to 2 for these participants to acquire a better fitting. Our approach yielded an average distance between head shape points and MRI skin surface of *M* = 1.62 (SD = 1.18) mm across participants.

Afterwards, we calculated the leadfield matrix using the coregistration model, source space, and BEM solution for each participant. The noise‐covariance matrix was estimated from epoched data using a period of 200 ms before the stimulus onset. Ledoit‐Wolf shrinkage was applied for regularization, and the alpha parameter was optimized through a cross‐validated search. The rank was inferred from the Maxwell filtering header.

Subject‐wise source reconstruction requires a forward model with rank estimation and a depth prior. As with the noise covariance matrix, ranks were inferred from the data. Additionally, the depth prior was estimated from the data using a weighting exponent of 0.8. We chose the Minimum Norm Estimate (MNE) as the inverse solution method, applying a loose orientation constraint (“weight” = 0.2; Lin et al. 2006). Pooling was performed by taking the norm of the loose orientations. In scenarios where reliable a priori information about source generators is uncertain—as is often the case in complex cognitive tasks—MNE is a valuable linear inverse method for projecting sensor measurements into source space (Hauk 2004). It estimates a source distribution with minimum (L2‐norm) current that best accounts for the measured data (Hämäläinen et al. 1993). In the time‐locked ERF analysis, the inverse operator was applied to the evoked data with an SNR of 3; while in the oscillatory power analysis, it was applied to the epoched data with an SNR of 1 (Gramfort et al. 2013; Lin et al. 2006). The SNR influences the regularization for scaling the noise‐covariance matrix.

#### 2.7.3 Inferential Statistics of the ERFs and Oscillatory Power

For the group‐level statistical comparisons, the individual estimated source activity or power spectral density of each condition was morphed to the average FreeSurfer brain template fsaverage (Fischl et al. 1999; Gramfort et al. 2013). This morphing transforms the source space of individual subjects into the same source space.

We computed the power spectral density of the inverse solution from epoched data using the multitaper method with discrete prolate spheroidal sequence (DPSS) windows and a bandwidth of 2 Hz. Cut‐offs of the frequency bands range from 4 to 7 Hz for theta, 8 to 12 Hz for alpha, 13 to 20 Hz for low beta, 21 to 29 Hz for high beta, and 30 to 42 Hz for gamma (Pavlov and Kotchoubey 2022).

We performed the group‐level statistic for the time‐locked ERFs and oscillatory power per frequency band during the two dual‐task phases (Figure 2) in two steps: We first tested for significant main effects of emotional expression, WM load, or a significant interaction of the two factors in a repeated measures analysis of variance (rmANOVA) using spatiotemporal (ERFs) or spatial (oscillatory power) one‐sided non‐parametric permutation‐based clustering (Maris and Oostenveld 2007) with 5000 permutations. Permutation‐based clustering is a mass‐univariate statistical approach that enables data‐driven statistical testing across spatial (each vertex) and temporal (each time point) dimensions while controlling for the family‐wise error (Maris and Oostenveld 2007). Traditional univariate ERP/ERF statistics, such as an ANOVA on mean or peak amplitudes at specific locations and within predefined time intervals, offer higher statistical sensitivity but provide limited temporal and spatial resolution of effects (Pernet et al. 2015; Groppe et al. 2011). A mass‐univariate approach is particularly advantageous when component latencies fluctuate due to experimental factors, such as complex stimulus material (Bentin et al. 1999). In permutation‐based clustering, a cluster is considered to have a significant effect if the sum of *F*‐values surpassed a predefined threshold—here the 95th percentile (α<0.05)—of the *F*‐value distribution in randomized data (Maris and Oostenveld 2007). To determine which group comparison differed significantly, we performed two‐sided post hoc *t*‐tests on the respective comparisons of the significant main or interaction effect with permutation‐based clustering as a second step. The analysis window was confined to the significant time window from the rmANOVA effect to reduce the number of samples along the temporal dimension while preserving spatiotemporal insights (Groppe et al. 2011). In case of a significant main effect, we averaged data over the other main effect and estimated the source activity as described above for the post hoc *t*‐statistics. Significant clusters were projected on a 3D brain (fsaverage) visualizing the statistical values (i.e., *F*‐values and *t*‐values) per cluster.

### 2.8 Eye‐Tracking and Behavioral Analysis

#### 2.8.1 Gaze Behavior

We processed the eye‐tracking data by interpolating gaps of missing samples in the time series. This was done using a cubic spline method, with a tolerance for maximum data loss of 75 samples. Subsequently, the time series were smoothed with a median‐based rolling window of 20 ms.

Pupil dilation was initially recorded using an arbitrary unit, defined as the number of pixels on the eye‐tracking camera that represented the pupil. To convert this measurement into millimeters, we measured an artificial eye with a known, fixed pupil area of 8 mm to obtain a scaling factor.

$$\text{Scaling factor}:\frac{8}{\sqrt{2107}}=0.174$$

and applied the scaling as follows:

$$\text{Pupil dilation in}\;\mathrm{mm}=\text{scaling factor}\times \sqrt{\text{pupil dilation}}\;\left(\mathrm{AU}\right)$$

While we partially compensated for drifts in the centered eye position at the start of each block, we observed a downward shift along the y‐axis over time. To counteract this bias, we applied a subject‐wise corrective offset to all trials. The offset was calculated for each subject by measuring the deviation between the screen center and the average y‐coordinate of fixations within a ring‐shaped region of interest (ROI) surrounding all squares during the n‐back encoding/retrieval phase. To reduce the influence of outliers, deviations exceeding 100 pixels were truncated, which affected 34.14% of cases. The average correction offset across subjects was *M* = 69.97 (SD = 31.52) pixels, which corresponds to *M* = 1.17° (SD = 0.53°) in visual angles. For the analyses, we used fixations detected by the SR Research online parser and extracted the following parameters: Mean fixation duration, the total count of fixation on the face ROI, and averaged pupil dilation during fixations during both dual‐task phases as well as the onset and total count of fixation on the square at the target position and of the target color during the n‐back phase. The ROI of the face stimulus was defined as a centered ellipse with a width radius of 5.93° and a height radius of 4.85° of visual angle (see Figure S6). The ROIs for the squares were defined as circles positioned around the square with a radius of 73 pixels (Figure S6). To account for family‐wise error across the 10 models, we corrected the α threshold for the statistical models using the Bonferroni method.

#### 2.8.2 Performance and Ratings

Behavioral data comprised performance measures of made errors in percentage (including false responses and misses) and reaction time (measured in seconds) during the trials, as well as the subjective ratings of perceived valence, arousal, and effort provided at the end of each block.

#### 2.8.3 Inferential Statistics of Gaze Behavior, Performance, and Ratings

We analysed the main effects of emotional expression and WM load as well as interactions on gaze behavior, task performance, and ratings using linear mixed models (Baayen et al. 2008; Bates et al. 2015) as implemented in the toolbox pymer4 (0.8.0; Jolly 2018). Data for each dependent variable were *z*‐standardized, and outliers exceeding the 95th percentile were removed. Additionally, we included the participant variable as random intercepts in the models to account for non‐systematic variations among individuals. The Satterthwaite approximation was applied to adjust the degrees of freedom for heteroscedasticity (using the “anova” method in lmer4; v. 1.1–14; Bates et al. 2015). As post hoc analyses of significant effects, we performed non‐parametric bootstrapping of group comparisons with 5000 iterations. Grand averages and their 95th percentile confidence intervals (CI) of the comparisons were plotted as boxplots. Comparable to the MEG analyses, comparisons with their mean's CI not including zero were considered significant (Cumming and Finch 2005).

## 3 Results

### 3.1 Behavioral Results of Performance and Subjective Ratings

Linear mixed models assessed whether WM load modulates a processing advantage of positive faces and a disadvantage of negative faces in facial emotion discrimination (Van Dillen and Koole 2009; van Dillen and Derks 2012; H.9–10d–e). For n‐back retrieval, they examined whether high WM load and negative faces deplete cognitive resources, increasing WM task interference compared to neutral and positive faces under high WM load (Van Dillen and Koole 2009; van Dillen and Derks 2012; H.17–18d–e). Model results are provided in detail in Table S1.

#### 3.1.1 Errors and Reaction Times

**FIGURE 3:**
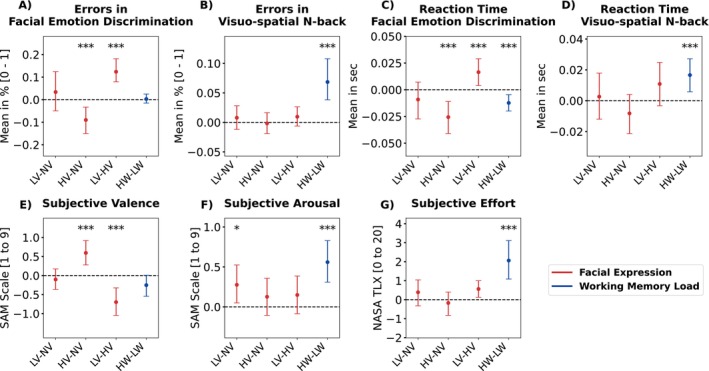
Post hoc comparisons of the main effects of emotional expression (red) and WM load (blue) for behavioural performance that is errors and reaction time as well as subjective valence, arousal and effort. Colored dots and error bars represent the bootstrapped grand averages and their Bonferroni‐corrected 2.5th and 97.5th confidence interval (CI) across participants. HV: high valence; HW: high WM load; LV: low valence; LW: low WM load; NV: neutral valence. Significance level from the linear mixed models: ***p<0.001, **p<0.01, *p<0.05.

Consistent with the subtask‐specific manipulation, we observed a significant main effect of emotional expression ($F_{\left(2,230\right)}$ = 24.52, *p* < 0.001) on discrimination errors and a significant main effect of WM load ($F_{\left(1,230\right)}$ = 68.27, *p* < 0.001) on retrieval errors in the n‐back subtask. In line with our hypothesis (H.1d), more errors were made during the discrimination of negative and neutral compared to positive faces (LV–HV: *M* = 0.124; 95% CI [0.079, 0.181]; HV–NV: *M* = −0.090; 95% CI [−0.151, −0.033]; Figure 3A). During the n‐back retrieval, high WM load in the 2‐back resulted in more errors compared to the 1‐back (HW–LW: *M* = 0.069; 95% CI [0.038, 0.108]; Figure 3B; H.13d). There was neither a significant main effect of WM load for emotion discrimination ($F_{\left(2,230\right)}$ = 0.05, *p* = 0.826; H.5d) nor a significant interaction in the subtasks (discrimination H.9d: $F_{\left(2,230\right)}$ = 0.30, *p* = 0.741; n‐back retrieval H.17d: $F_{\left(2,230\right)}$ = 0.01, *p* = 0.988).

Likewise, post hoc comparisons of a significant main effect of emotional expression ($F_{\left(2,230\right)}$ = 15.27, *p* < 0.001) revealed longer reaction times when discriminating negative and neutral compared to positive faces (LV–HV: *M* = 0.017; 95% CI [0.004, 0.029]; HV–NV: *M* = −0.026; 95% CI [−0.041, −0.011]; Figure 3C; H.2d). During the n‐back retrieval, we found a significant main effect of WM load ($F_{\left(1,230\right)}$ = 15.21, *p* < 0.001) with increased reaction times during high WM load (HW–LW: *M* = 0.017; 95% CI [0.006, 0.027]; Figure 3D; H.14d).

Surprisingly, a significant main effect of WM load ($F_{\left(1,230\right)}$ = 9.91, *p* = 0.002) indicated shorter reaction times during emotion discrimination, regardless of facial expression (HW–LW: *M* = −0.0123; 95% CI [−0.020, −0.005]; Figure 3C; H.6d). To examine whether this decline reflects a speed‐accuracy trade‐off under high WM load, we performed a follow‐up analysis. No significant relationship was observed between reaction time changes and accuracy under high WM load (Spearman rank correlation: $r_{s}$ = 0.107, *p* = 0.475). However, irrespective of WM load, reaction times were strongly correlated with discrimination errors ($r_{s}$ = 0.453, *p* < 0.001). Similar to the error results, there was no significant interaction between emotional expression and WM load on reaction time in the emotion discrimination ($F_{\left(2,230\right)}$ = 0.11, *p* = 0.898; H.10d) nor n‐back retrieval ($F_{\left(2,230\right)}$ = 0.28, *p* = 0.754; H.18d).

#### 3.1.2 Subjective Valence, Arousal, and Effort

The following linear mixed models analysed the effects of emotion expression (H.1–3e) and WM load (H.5–7,13e), as well as their interaction, on perceived overall valence, arousal, and effort across the block (H.9–10, 17e). There were significant main effects of WM load ($F_{\left(1,230\right)}$ = 6.27, *p* = 0.013) and emotional expression ($F_{\left(2,230\right)}$ = 18.80, *p* < 0.001) for valence (Figure 3E), significant main effects of WM load ($F_{\left(1,230\right)}$ = 41.08, *p* < 0.001) and emotional expression ($F_{\left(2,230\right)}$ = 3.33, *p* < 0.038) for arousal (Figure 3F), and a significant main effect of WM load ($F_{\left(1,230\right)}$ = 75.71, *p* < 0.001) for perceived effort (Figure 3G). There was no significant main effect of emotional expression on effort ratings ($F_{\left(2,230\right)}$ = 1.93, *p* = 0.148; Table S1; H.7e), nor were there significant interaction effects on valence, arousal, or effort ratings (Table S1; H.9–10e).

The post hoc analyses revealed higher valence ratings for positive compared to negative (LV–HV: *M* = −0.698; 95% CI [−1.048, −0.324]) and neutral expressions (HV–NV: *M* = 0.595; 95% CI [0.280, 0.919]; H.1e). There was no significant difference in rated valence scenarios with negative and neutral faces (H.1e). Valence ratings were lower for high compared to low WM load scenarios, indicating that facial expressions were generally perceived more negatively under high WM (HW–LW: *M* = −0.252; 95% CI [−0.544, 0.009]; n.s.; H.5e). Negative compared to neutral faces and high compared to low WM load led to increased subjective arousal (LV–NV: *M* = 0.276; 95% CI [0.050, 0.525]; H.2e; HW–LW: *M* = 0.560; 95% CI [0.310, 0.830]; H.6e).

Regarding the effort ratings, participants evaluated scenarios with high compared to low WM load to be more effortful (HW–LW: *M* = 2.062; 95% CI [1.087, 3.112]; H.7,13e). Scenarios with negative compared to positive faces were rated as more effortful (LV–HV: *M* = 0.562; 95% CI [0.124, 1.002]; ~H.3e); however, the main effect of emotional expression was not significant ($F_{\left(2,230\right)}$ = 1.93, *p* = 0.148).

In summary, both performance and subjective ratings were significantly affected by emotional expression and WM load. Increased cognitive demands were observed under high WM load and in scenarios with negative and partially neutral expressions. This was reflected in increased errors, longer reaction times, reduced valence, and increased arousal and effort.

### 3.2 MEG Results

We examined MEG activation differences between the emotional expression and WM load conditions, as well as their interaction, separately during both dual‐task phases (highlighted in Figure 2).

#### 3.2.1 ERFs of Emotional Face Processing

Permutation‐based spatiotemporal clustering revealed a significant main effect of emotional expression on ERF amplitudes during facial emotion discrimination ($p_{F-\text{statistic}}$ = 0.036) but not during n‐back encoding/retrieval, where the face was presented as a distractor for the second time (H.1,13a). We found no effect of WM load on face‐related ERFs (H.5a), nor an interaction between WM load and emotional expression (H.9–12a). The significant differences between the ERFs evoked by the different facial expressions started 190 ms after stimulus onset and lasted throughout the analysis time interval (1000 ms; Figure S2).

**FIGURE 4:**
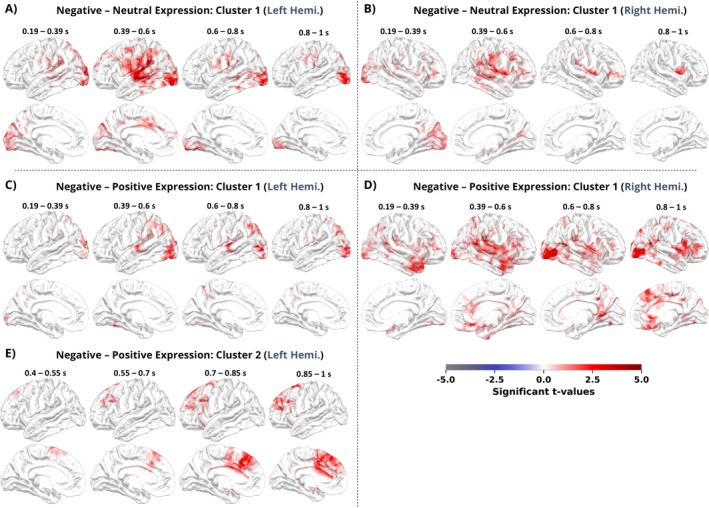
*T*‐statistic spatiotemporal clusters for significant comparisons of the main effect emotional expression. Contrasts between (A, B) negative and neutral emotional expressions and (C–E) negative and positive emotional expressions during facial emotion discrimination. *T*‐values from spatiotemporal clusters were grouped into four equally spaced time intervals and projected onto a 3D brain surface. Lateral (upper) and medial (lower) perspectives are visualized for each cluster and contrast.

To determine which emotional facial expressions modulate the ERFs and to examine their spatiotemporal distribution, we carried out post hoc comparisons with a *t*‐statistic‐based clustering (Figure 4). We observed significant clusters with increased ERFs and a similar temporally evolving spatial distribution when contrasting negative and neutral expressions (LV–NV; left hemisphere: *p* = 0.011; right hemisphere: *p* = 0.006; Figure 4A,B; H.3,4a) and negative and positive facial expressions (LV–HV; left hemisphere: *p* = 0.011; right hemisphere: *p* < 0.001; see Figure 4C,D). No ERF difference was found when contrasting positive and neutral facial expressions (H.2–4a).

In both contrasts, significant bilateral clusters started approximately at 190 ms after the onset of the face and persisted until the end of the presentation. Early significant differences were located at lateral occipital and parietal regions, including the posterior inferior parietal cortex, as well as right fronto‐temporal areas, including the anterior temporal lobe and OFC. Around 400–600 ms after stimulus onset, effects of pronounced ERF amplitudes for negative facial expressions were located in the lateral occipital cortex and ventral visual stream, temporo‐parietal cortex, the anterior insula, posterior superior temporal sulcus (STS), and anterior temporal lobe (compare to Figure 1A). From the medial view (Figure 4A,D, second row), we observed slightly increased activation in the caudal anterior and middle cingulate gyrus superior to the thalamus. These effects were greater in the right hemisphere when comparing negative with positive facial expressions (Figure 4D vs. 4C) and greater in the left hemisphere when comparing negative with neutral expressions (Figure 4A vs. 4B). In the final time interval from 800 to 1000 ms, we observed stronger evoked responses of the lateral occipital cortex and right IFG for negative compared to positive and neutral facial expressions (Figure 4A,C,D).

For the negative–positive expression contrast, we observed a second functional cluster on the left hemisphere, starting at a rather late time point around 400 ms after face onset (Figure 4E; *p* = 0.022). The cluster evolved strongest around the dlPFC, midfrontal gyrus, ACC, and premotor regions after 700 ms from stimulus onset until the end of the presentation.

In summary, our results show enhanced ERFs in the visual cortex and later in fronto‐temporal brain regions associated with emotional face processing and social cognition (see also Figure 1A) for negative but not positive facial expressions; and only when the emotionally negative faces were task‐relevant during the discrimination phase. This modulation was also not affected by the current WM load level (interaction effect; H.9–12a). An exploratory follow‐up analysis examining the relationships within and between cluster regions and time intervals can be found in Supporting Information A1.

#### 3.2.2 Oscillatory Power Modulations During the Visuo‐Spatial N‐Back Encoding/Retrieval Phase

To investigate the effect of WM load on oscillatory signatures (H.5–8, 13–16b) and its interactions with emotional distraction (H.9–11, 17–20b), spatial clustering was performed for the α, β, and θ frequency bands, as well as, on an exploratory basis, for the γ frequency band during the facial emotion discrimination (i.e., n‐back maintenance phase) and n‐back encoding/retrieval phase.

We observed significant main effects for WM load in the alpha (8–12 Hz; $p_{F-\text{statistic}}$ = 0.025; H.15–16b) and low beta (13–20 Hz; $p_{F-\text{statistic}}$ = 0.041; H.15b) frequency band during the n‐back encoding/retrieval phase. No significant spatial clusters were found in the theta, high beta, and gamma frequency bands. Additionally, there was no significant interaction effect was elicited by the emotional distractors (H.9–11, 17–20b). WM load level modulated oscillatory signatures only event‐locked during the encoding/retrieval WM phase (H.13b), but not during the maintenance of the encoded information (i.e., the facial emotion discrimination phase; H.5b).

**FIGURE 5:**
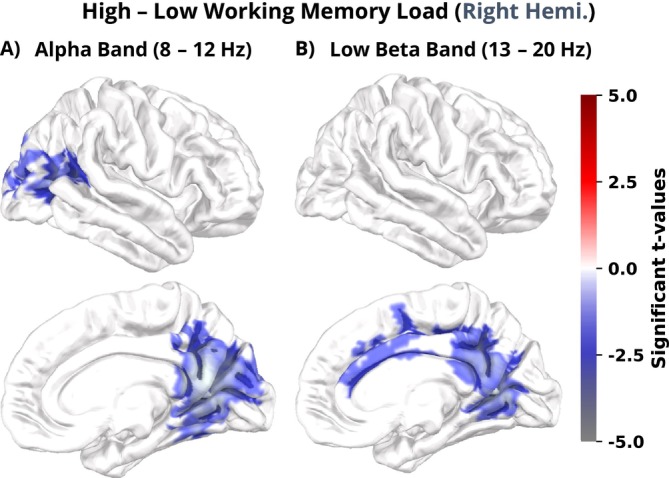
Significant *t*‐values of the alpha and low beta band cluster projected onto a 3D brain surface and visualized from both the lateral (upper row) and medial (lower row) perspectives.

The alpha frequency band cluster exhibited significantly reduced power during high compared to low WM load in the right middle and superior occipital gyrus, lingual gyrus, fusiform gyrus, retrosplenial cortex, and inferior margin of the precuneus ($p_{t-\text{statistic}}$ = 0.017; Figure 5A).

In the low beta band, we observed decreased oscillatory power during high WM load in the anterior lingual gyrus, inferior margin of the precuneus, inferior parietal lobule, SMA as well as the anterior and posterior cingulate gyrus ($p_{t-\text{statistic}}$ = 0.037; Figure 5B, see also Figure 1B).

Exploratory follow‐up analyses revealed relationships between the alpha band modulations and n‐back performance during high WM load. Modulation in the alpha band cluster was positively correlated with retrieval reaction time during high WM load (HW–LW alpha band cluster × HW reaction time: $r_{s}=.375$, *p* = 0.020; see Supporting Information A2, Table S3 and Figure S5 for details).

In conclusion, our MEG findings imply that the discrimination of emotional expressions modulated ERFs (H.1, 3–4a), while WM load influenced oscillatory brain responses in the alpha and low beta bands during the encoding and retrieval phase (H.13, 15–16b). Correlation analysis of oscillatory changes and n‐back performance indicates that decreased occipital alpha band power is associated with shorter retrieval reaction times but not with retrieval accuracy, under high WM load. ERFs and oscillatory modulations revealed different processing modes during the discrimination of negative faces as well as encoding and retrieval of WM under high load. Despite the absence of interaction or interference effects, the observed main effects are likely associated with increased demands on processing resources and attention during negative facial expression discrimination and high‐load visuo‐spatial WM.

### 3.3 Eye‐Tracking Results of the Fixations and Pupil Dilation

**FIGURE 6:**
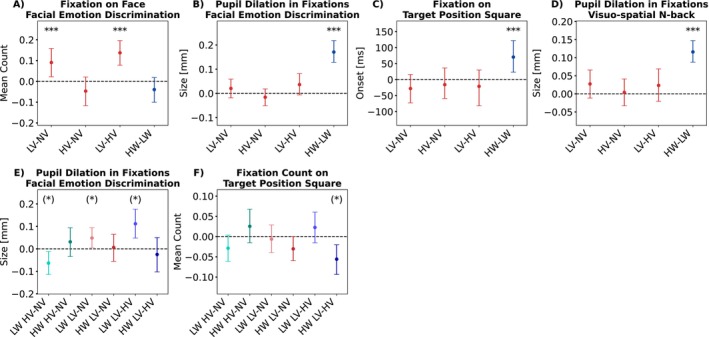
Post hoc comparisons of the main effects of emotional expression (red) and WM load (blue) of the gaze‐related fitted linear mixed models (A–D). Exploratory comparisons of interaction trends are depicted in (E) and (F). Colored dots and error bars represent the bootstrapped grand averages and their Bonferroni‐corrected 2.5th and 97.5th confidence interval (CI) across participants. Visuo‐spatial n‐back refers to the encoding/retrieval phase. HV: high valence; HW: high WM load; LV: low valence; LW: low WM load; NV: neutral valence. Significance level from the linear mixed models: ***p<0.001, **p<0.01, *p<0.05, () for significance level without Bonferroni correction of the fitted linear mixed models.

We examined interaction effects between emotional facial expressions and WM load on gaze behavior (i.e., fixation duration and count) and pupil dilation during the facial emotion discrimination (H.5–7c) and n‐back encoding/retrieval phase (H.17–18c) using linear mixed models. The statistics for all tested effects are provided in Table S4, and post hoc comparisons following a significant effect are illustrated in Figure 6.

#### 3.3.1 Effects During the Facial Emotion Discrimination Phase

We found a significant main effect of emotional expression on the total count of fixations within the ROI around the face during the emotion discrimination ($F_{\left(2,195\right)}$ = 8.17, *p* < 0.001; see Figure S6 for the display of ROIs in the fixation‐related analysis). Negative faces attracted more fixations than both neutral (LV–NV: *M* = 0.091; 95% CI [0.022, 0.158]; H.2c) and positive faces (LV–HV: *M* = 0.138; 95% CI [0.078, 0.196]; Figure 6A). There was no significant difference in the fixation count between positive and neutral expressions (HV–NV: *M* = −0.047; 95% CI [−0.117, 0.021]; H.2c), nor were there significant effects of WM load (H.6c) or an interaction with WM load (H.10c) on fixation count. Furthermore, no significant effects were observed for mean fixation duration on the face ROI (H.1,5,9c). In an exploratory follow‐up analysis of the main effect of emotional expression on fixation count, we examined which facial regions—the upper part including the eyes or the lower part including the mouth—attracted the most fixations. Results of a linear mixed model with the fixed effects face region (lower vs. upper half) × emotional expression (HV, NV, LV) revealed a significant difference in fixation counts between face regions ($F_{\left(1,195\right)}$ = 266.423, *p* < 0.001) and mean fixation duration ($F_{\left(1,195\right)}$ = 75.70, *p* < 0.001). More and longer fixations were positioned in the lower face region (lower–upper; count: *M* = 1.048; 95% CI [0.756, 1.321]; duration: *M* = 63.473 ms; 95% CI [35.994, 92.269]). There was no interaction between facial region and emotional expression (count: $F_{\left(1,195\right)}$ = 1.75, *p* = 0.176; duration: $F_{\left(1,195\right)}$ = 1.05, *p* = 0.352). Analyses including the task phase as a fixed effect in the linear mixed model are provided in Supporting Information A3.

##### 3.3.1.1 Sustained WM Load on Pupil Dilation

We observed a sustained WM load main effect on pupil dilation across the dual‐task ($F_{\left(1,195\right)}$ = 78.99, *p* < 0.001). Pupil dilation increased for high compared to low WM load during the facial emotion discrimination (HW–LW: *M* = 0.171 mm; 95% CI [0.128, 0.219]; Figure 6B; H.7c). There was no significant effect of emotional expression (H.3c) but a strong trend of interaction with WM load (H.11c) during the emotion discrimination ($F_{\left(2,195\right)}$ = 4.46, *p* < 0.013, n.s. after Bonferroni correction on linear mixed model level). Exploratory comparisons showed reduced pupil dilation for positive and increased pupil dilation for negative compared to neutral faces only during low WM load (LW HV–NV: *M* = −0.063 mm; 95% CI [−0.114, −0.011]; LW LV–NV: *M* = 0.048 mm; 95% CI [0.005, 0.094]; Figure 6E).

#### 3.3.2 Effects During the Visuo‐Spatial N‐Back Encoding/Retrieval Phase

During the n‐back phase, we observed a significant main effect of WM load on fixation onset at the target position ($F_{\left(1,176.25\right)}$ = 13.26, *p* < 0.001) and pupil dilation during fixations ($F_{\left(1,195\right)}$ = 16.65, *p* < 0.001; Figure 6C). During high WM load, the onset of fixation on the target position was delayed (HW–LW: *M* = 70.518 ms; 95% CI [23.253, 122.112]; H.14c) and pupil dilation was increased (HW–LW: *M* = 0.117 mm; 95% CI [0.088, 0.148]; Figure 6D; H.15c). There was a non‐significant trend of interaction between emotional expression and WM load on target fixation count ($F_{\left(2,195\right)}$ = 4.35; *p* < 0.014; H.17c). The exploratory comparisons revealed fewer fixations at the target position during high WM load for negative distractors compared to neutral (HW LV–NV: *M* = −0.030; 95% CI [−0.059, 0.000]; Figure 6F) and positive (HW LV–HV: *M* = −0.056; 95% CI [−0.093, −0.020]; Figure 6F) ones; while no differences were observed between expression conditions during low WM load. There were no significant effects on fixation measures within the face area (H.16,18c).

Taken together, the results of the fixation‐related correlates and pupil dilation indicated increased cognitive processing demand and required attention recruitment when discriminating negative facial expressions and under high WM load scenarios. Moreover, the WM load level induced by the n‐back subtask influenced pupil dilation during facial emotion discrimination, indicating a sustained WM dual‐task effect. Sustained WM load interacted with emotional expressions during the discrimination, eliminating differences in pupil dilation between emotional and neutral expressions under high WM load. During the n‐back encoding/retrieval phase, an interaction trend suggested that task‐oriented gaze behavior (i.e., fixation on the target position) was reduced for negative emotional distractors, but only under high WM load. Descriptive heatmaps of the fixations during the emotion discrimination and n‐back phase can be found in Figures S8 and S9.

## 4 Discussion

The objective of this whole‐head MEG study combined with eye‐tracking was to address the research gap in understanding the interaction effects of emotional facial expressions and varying WM load levels in a dual‐task paradigm. We aimed to identify the circumstances and mechanisms through which emotion and cognitive processing interact during emotion discrimination and visuo‐spatial WM. Additionally, we investigated brain mechanisms underlying the task processes and regulatory mechanisms triggered to mitigate indications of task interference when processing and attentional resources are depleted. We analyzed MEG source space activation, including ERFs linked to emotional face processing, oscillatory signatures associated with the inhibition of task‐irrelevant neural activity and WM load, as well as gaze‐related and behavioral responses during both dual‐task phases.

As hypothesized, negative facial expressions enhanced spatiotemporal ERFs during the emotion discrimination (Table 1; H.1, 3–4a). Importantly, our dual‐task study suggests that this effect involves top‐down mechanisms, as it was only observed when the emotional information was task‐relevant. High WM load decreased occipital alpha and parieto‐occipital low beta oscillations during the n‐back encoding and retrieval phase (Table 1; H.13, 15–16b). A WM load‐based decrease in occipital alpha power was associated with shorter retrieval time but not increased retrieval accuracy under high WM load. The relationship between the modulation and performance provides insight into the functional role of alpha band oscillation in enhancing information processing and attention allocation, but not WM maintenance, during increased load. Sustained cross‐task effects of high WM load were found on pupil size (H.7c), reaction time (H.6d), and perceived valence (H.5e). Our multimodal study presents converging physiological and behavioral evidence suggesting that the following processes are associated with greater attentional and processing demands: (1) discriminating negative compared to neutral and positive faces, which enhanced evoked brain responses linked to face processing and social cognition and increased pupil dilation, arousal, and effort; and (2) memory encoding and retrieval during high compared to low load, which decreased posterior alpha and low beta power and increased pupil dilation, arousal, and effort.

Trends for interactions between WM load and emotion of facial expression were present in both subtasks. During facial emotion discrimination, pupil dilation indicated a processing advantage for positive expressions (decrease) and a disadvantage for negative expressions (increase) during low WM load. However, these emotion‐specific processing differences disappeared under high WM load (H.9–11c–e). Moreover, during the n‐back encoding/retrieval phase, negative distractors reduced task‐related fixations on the target position but only during high WM load (H.17c). This effect may be associated with reduced attentional control and increased distractibility of negative faces during increased cognitive demands. The interpretation is further supported by the questionnaire result that participants rated blocks with negative facial expressions as more effortful to process (H.3e). By incorporating the neutral expression condition, we were able to extend existing knowledge (Van Dillen and Koole 2009; van Dillen and Derks 2012), demonstrating that interaction effects are not solely attributable to interference from negative faces, but also reflect a processing advantage for positive faces under low WM load.

However, we anticipated further interaction effects that our results did not support: During the emotion discrimination, high WM load was hypothesized to mitigate enhanced ERFs of emotional faces and to attenuate oscillatory signatures of WM maintenance (H.9–12a, b). During the n‐back encoding/retrieval phase under high WM load, we assumed that angry distractors would interfere with WM due to depleted cognitive resources and reduced attentional control (H.17–20a,b,d,e). This was expected to attenuate the oscillatory signatures of encoding and retrieval (H.17‐20b) and elicit enhanced ERFs of emotional face processing (H.17–20a). In the following, we discuss the observed effects in more detail and the context of previous work.

### 4.1 Processing and Discrimination Mechanisms of Emotional Facial Expressions

#### 4.1.1 Enhanced (Socio‐)Emotional Evaluation Processes for Negative Facial Expression Discrimination

Negative facial expressions modulated spatiotemporal ERF with significantly increased activation in fronto‐temporal brain networks, the insula–ACC complex, and occipital regions, including the secondary and extrastriate visual cortex. This ERF modulation was only observed during the emotion discrimination and only for negative but not positive faces (H.1,3–4a).

We propose that the observed spatiotemporal ERF clusters may be divided into two functional components. This aligns with the dissociation theory of Ochsner and Gross (2005), who suggest a ventral stream for emotion processing and a dorsal stream for emotion regulation. In our study, the first cluster comprises increased evoked amplitudes in regions associated with emotional face processing (Vuilleumier et al. 2003; Vuilleumier and Pourtois 2007; Winston et al. 2003; Posamentier and Abdi 2003; see Figure 1). These enhanced activation patterns of the cluster are observed when contrasting negative to positive but also neutral facial expressions. The engagement of the insula and late reactivation of the occipital cortex suggest that this cluster is also functionally related to more intense processing of negative facial expressions (Carr et al. 2003; Haxby et al. 2000; Montgomery and Haxby 2008; Pitcher et al. 2008; Said et al. 2011). The intense processing of negative faces is also reflected in increased fixation counts and pupil dilation during the discrimination of negative expressions.

We found a second cluster that appeared only when contrasting negative with positive facial expressions. Since it comprises lateral prefrontal areas and the ACC, this cluster may be linked to goal‐directed regulatory mechanisms to overcome potential interference and disrupt lasting effects of processing and discriminating negative facial expressions Ochsner and Gross (2005); García‐Pacios et al. (2017). This explanation is supported by recent findings from Dong et al. (2024). In their MEG study, negative image distractors increased activation in the insula during the early phase of the P3b followed by increased activation in the ACC. They associated this pattern with attentional control to suppress the effect of the distracting negative stimuli.

Although this enhanced ERF linked to attentional control was not modulated by WM load, pupil dilation revealed an interaction between WM load and emotion‐specific processing for positive and negative expressions. The effect on pupil dilation aligns with the second ERF cluster and previous findings (Van Dillen and Koole 2009; van Dillen and Derks 2012) suggesting processing advantages for positive faces and disadvantages for negative faces under low cognitive demand. However, under high WM load, emotion‐specific processing in pupil dilation disappeared (Van Dillen and Koole 2009; van Dillen and Derks 2012). This finding suggests that the threshold for detecting an interaction between emotion and WM load may be higher at the neural level than in gaze‐related measures and points out the importance of integrating physiological and behavioral measures.

#### 4.1.2 Discrimination Advantage for Positive Facial Expressions

Based on the second ERF cluster and interaction trend in pupil dilation, it can be concluded that negative face processing was enhanced and linked to increased cognitive demands, while positive faces exhibited a processing advantage—at least under low WM load. This is consistent with previous research (Becker et al. 2011; Bucher and Voss 2019; Calvo and Beltrán 2013; Weidner et al. 2024; Van Dillen and Koole 2009). In comparing only negative and positive facial expressions, Van Dillen and Koole (2009) attributed this difference solely to a negativity bias toward angry faces. However, by including a neutral reference, we could show performance advantages—in the form of reduced errors and reaction time—during the discrimination of positive compared to neutral and negative faces but not negative compared to neutral faces. In the ongoing debate on whether there is a processing advantage for negative or positive expressions in visual attention, Xu et al. (2021) concluded in their review that tasks explicitly focusing on emotion processing of photorealistic faces tend to show an advantage for positive expressions. Horstmann et al. (2012) offered a stimulus‐level explanation for the processing advantage. The authors demonstrated that the visibility of teeth in photorealistic emotional faces facilitates the search in the face‐in‐a‐crowd paradigm. Given that teeth are visible only in positive facial expressions in the FACES database, participants in our study could have exploited this information for efficient discrimination when distinguishing positive from negative and neutral faces. In line with this, Jehna et al. (2011) reported that neutral faces were more likely to be misjudged as negative than positive faces (see also Lee et al. 2008; Weidner et al. 2024; Albohn et al. 2019). Our findings of increased fixations on the lower face ROI support the conclusion that participants relied on information from the mouth region for discrimination. A supplementary fixation analysis, including task phase as a factor, further suggests that this strategy was specific to emotion discrimination and did not occur in the n‐back retrieval/encoding phase (see Figure S7). Interestingly, the potential exploitation of low‐level facial characteristics appeared to have a minimal impact on the neural processing in our study: We did not observe modulated spatiotemporal ERFs when contrasting neutral and positive facial expressions, despite their difference in low‐level features.

#### 4.1.3 Template‐Based Face Encoding as Emotion Discrimination Strategy

Our behavioral and eye‐tracking results, thus, support the extension of the prototype‐referenced shape encoding theory (Leopold et al. 2001, 2006) to facial expression discrimination (Skinner and Benton 2010). Hence, behavioral and gaze‐related differences between positive and negative facial expressions arise from their differing configural resemblance to the internal template, which is neutral faces (Skinner and Benton 2010). While positive faces triggered early and efficient discrimination due to strong configural differences to the template, negative faces might have exhibited a higher similarity to neutral faces, resulting in ambiguity during discrimination (see also the discussion of Weidner et al. 2024). Hence, they required a more thorough examination, which involved recruiting additional neural processes, until clear discriminative features were identified. This would place a greater toll on cognitive resources. The significantly increased number of fixations and ACC and lateral PFC activation further indicate enhanced attentional engagement with negative faces and the need for control mechanisms to disengage after discrimination (Becker and Rheem 2020; Eastwood et al. 2003; Horstmann et al. 2006). To summarize, from a template‐based facial encoding perspective, the greatest difference in encoding and discrimination fluency can be expected for negative compared to positive facial expressions; at least during low WM load. The latter is associated with efficient and resource‐saving discrimination, whereas negative facial expressions appear to be resource‐demanding.

The interaction trend in pupil dilation further supports the involvement of top‐down attentional mechanisms to discriminate facial expressions. Unlike automatic processes, such deliberate mechanisms can be influenced by the current WM load and available cognitive resources (Schindler and Bublatzky 2020). It appears that explicit instruction toward emotion classification can further influence whether processing is deliberate or automatic—particularly for positive facial expressions (Xu et al. 2021; Rellecke et al. 2012). This aligns with the absence of any early enhanced ERFs by emotional expressions in our study. Contrary to previous literature (Eimer and Holmes 2007; Schupp et al. 2006), we did not observe enhanced ERFs for positive facial expressions. One explanation is provided by Almeida et al. (2016), who demonstrated a positive relationship between the modulation of face‐related M/EEG components and perceived arousal. Since participants in our study rated angry—but not happy—faces more arousing than neutral ones, the amplified ERFs may be at least partially driven by arousal. In addition to the heightened arousal elicited by angry faces, the increased evolutionary relevance of negative facial expressions, along with their communicative function in signaling potential threat and initiating adaptive responses in social contexts, may also account for the observed processing differences (Adolphs 2003).

### 4.2 Effects of Cognitive Capacity Limits During High WM Load

Another key finding of our study was a significant main effect of WM load, with high load decreasing oscillatory alpha and low beta band power in posterior regions (H.13, 15–16b) and increasing pupil dilation (H.15c), fixation speed (H.14c), the number of errors (H.13d) and reaction times (H.14d) during the encoding and retrieval phase of the visuo‐spatial WM n‐back subtask. Furthermore, WM load level affected the overall effort, arousal, and even valence experienced across the block (H.5–7, 13e). A decline in occipital alpha band power and its WM load‐based modulation was associated with faster retrieval reaction times but not retrieval accuracy under high WM load (Supporting Information Analysis A2).

The WM load effects on performance are in line with previous studies (e.g., Leung et al. 2004; Schroeder et al. 2018) and can be explained by insufficient capacity and limited resources to process and maintain task‐relevant information.

At the brain level, increased WM load reduced posterior alpha and low beta band power in inferior parietal regions, which strongly overlap with the WM brain network and load‐modulated areas (e.g., Lamm et al. 2001; Michels et al. 2010; Mitchell et al. 2007; see also Figure 1B). In our study, frontal theta power did not increase with increasing WM load (cf. Jensen and Tesche 2002; Costers et al. 2020; H.6,14b). A possible explanation is that frontal theta signals primarily originate from superficial radial dipole layers, which are reliably detected by EEG but less effectively captured by MEG (Srinivasan et al. 2006).

Modulations in alpha band oscillations have been proposed to guide information processing (Jokisch and Jensen 2007; Klimesch et al. 2007; Schroeder et al. 2018) with increased alpha power indicating top‐down suppression of neural activity and decreased alpha power indicating cortical engagement. Hence, our results suggest increased visual processing and involvement of the temporo‐occipital regions during high compared to low WM load scenarios. An explanation for this phenomenon is that visual processing is enhanced to accommodate the heightened cognitive demand of the more challenging task. This notion is further supported by our correlation results (Supporting Information Analysis A2), which demonstrate that greater decreases in occipital alpha power are associated with shorter retrieval times under high WM load. Contrary to our hypothesis (H.16b), we conclude that the modulation of occipital alpha band power likely reflects a compensatory mechanism that enhances cortical engagement, facilitating attentional allocation and information processing under high WM load (Jokisch and Jensen 2007; Klimesch et al. 2007; Schroeder et al. 2018).

The interpretation of load effects on beta oscillatory power is still debated in the literature (Pavlov and Kotchoubey 2022). Previous studies have proposed a link between increased beta oscillations and WM maintenance (e.g., Chen and Huang 2015; Deiber et al. 2007; Engel and Fries 2010). Consistent with this suggestion, we observed a decrease in low beta oscillatory power in key regions associated with WM and spatial cognition (Figure 1B), including the inferior parietal areas, retrosplenial cortex, and inferior precuneus (Vann et al. 2009; Mitchell et al. 2018). We propose that modulatory changes in parieto‐occipital low beta band power are related to WM maintenance and the decay of task‐relevant information during high load (Chen and Huang 2015; Deiber et al. 2007; Engel and Fries 2010; Kopell et al. 2011; Salazar et al. 2012).

To conclude, there is a partial spatial overlap of modulations in the inferior parietal areas between the alpha and low beta band clusters. However, the alpha band cluster includes more temporo‐occipital regions, while the low beta band cluster contains almost exclusively inferior parietal regions, which reflect posterior key areas associated with WM (Figure 1B). The slightly different spatial patterns and the relationship between alpha band modulations and retrieval performance indicate distinct functional roles of band‐specific oscillations in the visuo‐spatial WM encoding and retrieval. Decreased occipital alpha power likely represents a compensatory mechanism to enhance information processing under high WM load (Jokisch and Jensen 2007; Klimesch et al. 2007; Schroeder et al. 2018), whereas reduced parietal low beta band power may reflect diminished WM maintenance and the decay of encoded information (Chen and Huang 2015; Deiber et al. 2007; Engel and Fries 2010; Salazar et al. 2012; Kopell et al. 2011).

#### 4.2.1 Sustained WM Load Effects due to Increased Cognitive Demand

Contrary to our initial hypotheses, we did not observe oscillatory modulations locked to the onset of the facial stimuli during emotion discrimination (H.5–8b) nor interactions with facial expressions during both subtasks (H.9–11, 17–20b). However, we observed different processing modes reflecting a higher demand on cognitive resources: Negative facial expressions recruited additional neural regions associated with social cognition and categorization as well as amplified activation in the emotional face brain network. High WM load led to decreased WM maintenance and compensatory engagement of occipital regions to enhance attention allocation and information processing.

Due to a greater demand on processing resources, we observed a sustained WM load effect across subtasks: Pupil dilation increased for positive and decreased for negative faces, while reaction time decreased during emotion discrimination with concurrent high compared to low WM load. This pattern suggests that the emotional expression effect on gaze behavior is reduced during high WM load (see Van Dillen and Koole 2009 for similar behavioral findings). The faster reaction times during high compared to low WM load pointed to quick decision‐making in emotion discrimination, presumably driven by enhanced processing resource allocation for the upcoming encoding and retrieval of information from WM.

### 4.3 How Robust Are Interaction Effects?

Although some studies have reported interactions between emotion and WM load on neurophysiological and behavioral signatures (e.g., van Dillen and Derks 2012; MacNamara et al. 2012; García‐Pacios et al. 2015a), we could not entirely replicate these effects in this dual‐task study (H.9–12; 17–20a,b). Given the inconsistency in detecting interaction effects in the literature and relatively small reported effect sizes (Brockhoff et al. 2022; Schweizer et al. 2019), some interaction effects may not be robust to variations in experimental design or stimulus material.

A novel aspect less examined in previous studies was the contextual change in task relevance of facial stimuli, specifically their emotional expressions, in our dual‐task. Contrary to van Dillen and Derks (2012), we aimed to amplify the effect of emotional negative and positive expressions by using a task that explicitly indicated emotion (Rellecke et al. 2012). However, this may have had the opposite effect in the n‐back encoding/retrieval phase, reducing distraction and interference during the second presentation of the same, now task‐irrelevant face due to prior processing (also discussed by Tavares et al. 2016).

Further, we cannot exclude the possibility that not only emotion effects on ERFs but also the interaction with WM load are partly influenced by stimulus arousal. This is particularly relevant as most studies did not report subjective ratings (but cf. García‐Pacios et al. 2015a). Britton et al. (2006) compared valence and arousal ratings for emotional facial stimuli and images (International Affective Picture System; Lang et al. 1997). The IAPS dataset has also been used in studies showing interaction effects for WM and negative stimuli (García‐Pacios et al. 2015a; Tavares et al. 2016 for behavioral effects). Compared to emotional faces, images were rated significantly more positive for depicted happy and neutral emotions, more negative for negative emotions, and more arousing across all emotion categories (Britton et al. 2006). Based on these findings, faces might be considered weaker distractors due to their lower arousal strength (Britton et al. 2006; Ochsner and Gross 2005; Tavares et al. 2016). However, Carretié et al. (2012) compared emotional faces and images in their potential to capture attention and observed similar behavioral and neurophysiological responses. Nevertheless, the arousal intensity of the stimuli may have influenced the likelihood of detecting an interaction during discrimination. In addition, arousal levels may reach a ceiling earlier for emotional facial stimuli than for non‐facial images (Britton et al. 2006). Future studies should systematically examine the impact of arousal and stimulus type on interaction effects.

### 4.4 Limitation and Future Directions

The use of the naturalistic FACES database impacted the interpretation of certain effects. We decided against using schematic or morphed emotional faces, which are restricted to an oval shape to remove additional distinctive features, such as hair. However, naturalistic faces carry the risk of introducing confounding low‐level features. While these features are negligible when evenly distributed across expressions, they may bias emotion discrimination if they systematically indicate a specific expression (e.g., visible teeth in happy faces). Therefore, our findings of a template‐based discrimination strategy should be replicated using schematic faces with carefully controlled low‐level features.

As our sample resembled young, highly educated students, the generalizability of the findings to other age groups may be limited.

Concerning source localization, MNE relies on linear assumptions about the relationship between the electrical activity of the brain and measured signals (Hämäläinen et al. 1993). Interactions of activity from brain regions nearby might not be accurately captured. It also tends to favor sources closer to the sensors when solving the inverse problem (Hämäläinen et al. 1993). By adding a regularization to capture deeper sources such as insular activation, the estimates obtained sometimes have a spatial spread that is scattered around the actually smaller underlying deep sources.

Our study did not explore individual variations in executive functions, cognitive control over emotionally irrelevant information, or WM capacity. Future research should examine how these individual differences influence the interplay between emotion and cognition in the dual‐task (e.g., Dolcos and Denkova 2014; Dolcos et al. 2011). Linking such variations to neural correlates when facing an emotion‐cognition interaction could potentially also serve as diagnostic indices from a clinical perspective (Schweizer et al. 2019).

In future investigations, integrating eye‐tracking with MEG to perform fixation‐related analyses aligned with the onset of fixation on emotional stimuli may enhance sensitivity in detecting time‐locked interaction effects (Baccino and Manunta 2005; Spiering and Dimigen 2024). Furthermore, additional research could include transcranial magnetic stimulation in the emotion‐cognition dual‐task to increase the likelihood of task interference by inhibiting regulatory mechanisms (Olk et al. 2015).

## 5 Conclusion

In a dual‐task, we investigated spatiotemporal and oscillatory signatures of emotional face processing and visuo‐spatial WM load. Our MEG findings revealed enhanced ERFs that were spatially located across the insula, ACC, and face‐specific occipital regions during the discrimination of negative facial expressions but not during the encoding and retrieval of n‐back information from WM. Hence, negative faces amplified face processing and social cognition only when they were task‐relevant. Furthermore, when contrasting negative and positive facial expressions, enhanced ERFs were observed in prefrontal regions and the ACC at late time intervals of the discrimination. Both areas are associated with goal‐directed executive functions and may be linked to attentional control, facilitating the dissociation from negative faces to prevent interference with the WM subtask. In addition to these findings, task performance and gaze behavior demonstrated an advantage in discriminating positive faces, indicated by fewer errors and fixations on the face area as well as reduced reaction times and subjective effort, and decreased fixations on the face area. An interaction trend in pupil dilation suggested that the processing advantage for positive faces, along with a disadvantage for negative faces, diminished with increasing WM load.

In the n‐back encoding/retrieval phase, high WM load attenuated alpha band power in temporo‐occipital regions, while low beta band power was reduced in parieto‐occipital regions and the cingulate cortex. The spatial localisation of the clusters and their association with retrieval performance suggest that alpha and beta oscillations have different functional roles, during increased WM load. Reduced temporo‐occipital alpha power indicates compensatory cortical engagement to enhance attention allocation and information processing, while inferior parietal low‐beta power is proposed to be associated with WM maintenance. At the behavioral level, an interaction trend was observed for negative distractors with reduced fixations on the target position, but only during high WM load. Sustained effects of increased WM load were observed in task performance and gaze behavior across both subtasks. High WM load reduced the perceived valence of facial expressions and increased pupil size and reaction time during both subtasks.

In conclusion, the study advanced our understanding of when and how negative faces influence ERFs, mechanisms underlying emotion discrimination in naturalistic faces, interaction effects in gaze behavior, and the role of WM‐related oscillatory alpha and low beta power under different load levels.

## Author Contributions

Conceptualization: K.L., J.W.R.; Methodology: K.L., J.W.R.; Investigation: K.L.; Formal analysis: K.L.; Writing (original draft, review and editing): K.L., J.W.R.; Funding Acquisition: K.L., J.W.R.; Supervision: J.W.R.

## Ethics Statement

The study was pre‐registered on the Open Science Framework (https://osf.io/cg7nm) and the protocol was approved by the ethics committee of the Carl von Ossietzky University, Oldenburg, Germany (Ref. EK/2018/070).

## Consent

Participants provided informed consent following the Declaration of Helsinki. They were informed that their participation was voluntary, that they could withdraw at any time during the experiment, and that they could request the deletion of their data up until the point of anonymization. The informed consent also included a section on the publication of anonymized data and aggregated results.

## Conflicts of Interest

The authors declare no conflicts of interest.

## Supporting information


**Data S1** Supporting Information.

## Data Availability

Data supporting the findings of this manuscript will be made available on request via the OSF project (https://osf.io/um6vw/).

## References

[hbm70242-bib-0001] Adolphs, R. 2003. “Cognitive Neuroscience of Human Social Behaviour.” Nature Reviews Neuroscience 4: 165–178. 12612630 10.1038/nrn1056

[hbm70242-bib-0002] Albohn, D. N. , J. C. Brandenburg , and R. B. Adams . 2019. “Perceiving Emotion in the “Neutral” Face: A Powerful Mechanism of Person Perception.” In The Social Nature of Emotion Expression, Springer eBook Collection, edited by U. Hess and S. Hareli , 25–47. Springer.

[hbm70242-bib-0003] Almeida, P. R. , F. Ferreira‐Santos , P. L. Chaves , T. O. Paiva , F. Barbosa , and J. Marques‐Teixeira . 2016. “Perceived Arousal of Facial Expressions of Emotion Modulates the n170, Regardless of Emotional Category: Time Domain and Time–Frequency Dynamics.” International Journal of Psychophysiology 99: 48–56. 26659012 10.1016/j.ijpsycho.2015.11.017

[hbm70242-bib-0004] Baayen, R. H. , D. J. Davidson , and D. M. Bates . 2008. “Mixed‐Effects Modeling With Crossed Random Effects for Subjects and Items.” Journal of Memory and Language 59: 390–412.

[hbm70242-bib-0005] Baccino, T. , and Y. Manunta . 2005. “Eye‐Fixation‐Related Potentials: Insight Into Parafoveal Processing.” Journal of Psychophysiology 19: 204–215.

[hbm70242-bib-0006] Baddeley, A. 1992. “Working Memory.” Science 255: 556–559. 1736359 10.1126/science.1736359

[hbm70242-bib-0007] Baddeley, A. 1996. “The Fractionation of Working Memory.” Proceedings of the National Academy of Sciences of the United States of America 93: 13468–13472. 8942958 10.1073/pnas.93.24.13468 PMC33632

[hbm70242-bib-0008] Bates, D. , M. Mächler , B. Bolker , and S. Walker . 2015. “Fitting Linear Mixed‐Effects Models Using lme4.” Journal of Statistical Software 67: 1–48.

[hbm70242-bib-0009] Becker, D. V. , U. S. Anderson , C. R. Mortensen , S. L. Neufeld , and R. Neel . 2011. “The Face in the Crowd Effect Unconfounded: Happy Faces, Not Angry Faces, Are More Efficiently Detected in Single‐ and Multiple‐Target Visual Search Tasks.” Journal of Experimental Psychology: General 140: 637–659. 21744984 10.1037/a0024060

[hbm70242-bib-0010] Becker, D. V. , and H. Rheem . 2020. “Searching for a Face in the Crowd: Pitfalls and Unexplored Possibilities.” Attention, Perception, & Psychophysics 82: 626–636. 10.3758/s13414-020-01975-7 32043216

[hbm70242-bib-0011] Bentin, S. , and L. Y. Deouell . 2000. “Structural Encoding and Identification in Face Processing: Erp Evidence for Separate Mechanisms.” Cognitive Neuropsychology 17: 35–55. 20945170 10.1080/026432900380472

[hbm70242-bib-0012] Bentin, S. , Y. Mouchetant‐Rostaing , M.‐H. Giard , J.‐F. Echallier , and J. Pernier . 1999. “Erp Manifestations of Processing Printed Words at Different Psycholinguistic Levels: Time Course and Scalp Distribution.” Journal of Cognitive Neuroscience 11: 235–260. 10402254 10.1162/089892999563373

[hbm70242-bib-0013] Bonnefond, M. , and O. Jensen . 2012. “Alpha Oscillations Serve to Protect Working Memory Maintenance Against Anticipated Distracters.” Current Biology 22: 1969–1974. 23041197 10.1016/j.cub.2012.08.029

[hbm70242-bib-0014] Boonstra, T. W. , T. Y. Powell , S. Mehrkanoon , and M. Breakspear . 2013. “Effects of Mnemonic Load on Cortical Activity During Visual Working Memory: Linking Ongoing Brain Activity With Evoked Responses.” International Journal of Psychophysiology: Official Journal of the International Organization of Psychophysiology 89: 409–418. 23583626 10.1016/j.ijpsycho.2013.04.001

[hbm70242-bib-0015] Bradley, M. M. , and P. J. Lang . 1994. “Measuring Emotion: The Self‐Assessment Manikin and the Semantic Differential.” Journal of Behavior Therapy and Experimental Psychiatry 25: 49–59. 7962581 10.1016/0005-7916(94)90063-9

[hbm70242-bib-0016] Britton, J. C. , S. F. Taylor , K. D. Sudheimer , and I. Liberzon . 2006. “Facial Expressions and Complex Iaps Pictures: Common and Differential Networks.” NeuroImage 31: 906–919. 16488159 10.1016/j.neuroimage.2005.12.050

[hbm70242-bib-0017] Brockhoff, L. , S. Schindler , M. Bruchmann , and T. Straube . 2022. “Effects of Perceptual and Working Memory Load on Brain Responses to Task‐Irrelevant Stimuli: Review and Implications for Future Research.” Neuroscience and Biobehavioral Reviews 135: 104580. 35189162 10.1016/j.neubiorev.2022.104580

[hbm70242-bib-0018] Bucher, A. , and A. Voss . 2019. “Judging the Mood of the Crowd: Attention Is Focused on Happy Faces.” Emotion 19: 1044–1059. 30265079 10.1037/emo0000507

[hbm70242-bib-0019] Cairo, T. A. , P. F. Liddle , T. S. Woodward , and E. T. C. Ngan . 2004. “The Influence of Working Memory Load on Phase Specific Patterns of Cortical Activity.” Cognitive Brain Research 21: 377–387. 15511653 10.1016/j.cogbrainres.2004.06.014

[hbm70242-bib-0020] Calvo, M. G. , and D. Beltrán . 2013. “Recognition Advantage of Happy Faces: Tracing the Neurocognitive Processes.” Neuropsychologia 51: 2051–2061. 23880097 10.1016/j.neuropsychologia.2013.07.010

[hbm70242-bib-0021] Cao, F. , K. Zeng , W. Li , et al. 2022. “Influence of Scene‐Based Expectation on Facial Expression Perception: The Moderating Effect of Cognitive Load.” Biological Psychology 168: 108247. 34968555 10.1016/j.biopsycho.2021.108247

[hbm70242-bib-0022] Carr, L. , M. Iacoboni , M.‐C. Dubeau , J. C. Mazziotta , and G. L. Lenzi . 2003. “Neural Mechanisms of Empathy in Humans: A Relay From Neural Systems for Imitation to Limbic Areas.” Proceedings of the National Academy of Sciences of the United States of America 100: 5497–5502. 12682281 10.1073/pnas.0935845100 PMC154373

[hbm70242-bib-0023] Carretié, L. , D. Kessel , A. Carboni , et al. 2012. “Exogenous Attention to Facial vs Non‐Facial Emotional Visual Stimuli.” Social Cognitive and Affective Neuroscience 8: 764–773. 22689218 10.1093/scan/nss068 PMC3791067

[hbm70242-bib-0024] Chaumon, M. , D. V. Bishop , and N. A. Busch . 2015. “A Practical Guide to the Selection of Independent Components of the Electroencephalogram for Artifact Correction.” Journal of Neuroscience Methods 250: 47–63. 25791012 10.1016/j.jneumeth.2015.02.025

[hbm70242-bib-0025] Chen, X. , E. Sorenson , and K. Hwang . 2023. “Thalamocortical Contributions to Working Memory Processes During the n‐Back Task.” Neurobiology of Learning and Memory 197: 107701. 36435360 10.1016/j.nlm.2022.107701 PMC9805524

[hbm70242-bib-0026] Chen, Y. , and X. Huang . 2015. “Modulation of Alpha and Beta Oscillations During an n‐Back Task With Varying Temporal Memory Load.” Frontiers in Psychology 6: 2031. 26779113 10.3389/fpsyg.2015.02031 PMC4705233

[hbm70242-bib-0027] Collins, D. L. , P. Neelin , T. M. Peters , and A. C. Evans . 1994. “Automatic 3d Intersubject Registration of Mr Volumetric Data in Standardized Talairach Space.” Journal of Computer Assisted Tomography 18: 192–205. 8126267

[hbm70242-bib-0028] Costers, L. , J. Van Schependom , J. Laton , et al. 2020. “Spatiotemporal and Spectral Dynamics of Multi‐Item Working Memory as Revealed by the n‐Back Task Using Meg.” Human Brain Mapping 41: 2431–2446. 32180307 10.1002/hbm.24955 PMC7267970

[hbm70242-bib-0029] Cowan, N. 2005. “Working Memory Capacity.” In Essays in Cognitive Psychology. Psychology Press.

[hbm70242-bib-0030] Cowan, N. 2017. “The Many Faces of Working Memory and Short‐Term Storage.” Psychonomic Bulletin & Review 24: 1158–1170. 27896630 10.3758/s13423-016-1191-6

[hbm70242-bib-0031] Cromheeke, S. , and S. C. Mueller . 2014. “Probing Emotional Influences on Cognitive Control: An Ale Meta‐Analysis of Cognition Emotion Interactions.” Brain Structure & Function 219: 995–1008. 23563751 10.1007/s00429-013-0549-z

[hbm70242-bib-0032] Cumming, G. , and S. Finch . 2005. “Inference by Eye: Confidence Intervals and How to Read Pictures of Data.” American Psychologist 60: 170–180. 15740449 10.1037/0003-066X.60.2.170

[hbm70242-bib-0033] Dale, A. M. , B. Fischl , and M. I. Sereno . 1999. “Cortical Surface‐Based Analysis. i. Segmentation and Surface Reconstruction.” NeuroImage 9: 179–194. 9931268 10.1006/nimg.1998.0395

[hbm70242-bib-0034] Deffke, I. , T. Sander , J. Heidenreich , et al. 2007. “MEG/EEG Sources of the 170‐ms Response to Faces Are Co‐Localized in the Fusiform Gyrus.” NeuroImage 35: 1495–1501. 17363282 10.1016/j.neuroimage.2007.01.034

[hbm70242-bib-0035] Deiber, M.‐P. , P. Missonnier , O. Bertrand , et al. 2007. “Distinction Between Perceptual and Attentional Processing in Working Memory Tasks: A Study of Phase‐Locked and Induced Oscillatory Brain Dynamics.” Journal of Cognitive Neuroscience 19: 158–172. 17214572 10.1162/jocn.2007.19.1.158

[hbm70242-bib-0036] Desikan, R. S. , F. Ségonne , B. Fischl , et al. 2006. “An Automated Labeling System for Subdividing the Human Cerebral Cortex on MRI Scans Into Gyral Based Regions of Interest.” NeuroImage 31: 968–980. 16530430 10.1016/j.neuroimage.2006.01.021

[hbm70242-bib-0037] D'Esposito, M. , and B. R. Postle . 2015. “The Cognitive Neuroscience of Working Memory.” Annual Review of Psychology 66: 115–142. 10.1146/annurev-psych-010814-015031 PMC4374359 25251486

[hbm70242-bib-0038] Dolan, R. J. 2002. “Emotion, Cognition, and Behavior.” Science 298: 1191–1194. 12424363 10.1126/science.1076358

[hbm70242-bib-0039] Dolcos, F. , and E. Denkova . 2014. “Current Emotion Research in Cognitive Neuroscience: Linking Enhancing and Impairing Effects of Emotion on Cognition.” Emotion Review 6: 362–375.

[hbm70242-bib-0040] Dolcos, F. , A. D. Iordan , and S. Dolcos . 2011. “Neural Correlates of Emotion‐Cognition Interactions: A Review of Evidence From Brain Imaging Investigations.” Journal of Cognitive Psychology 23: 669–694. 22059115 10.1080/20445911.2011.594433 PMC3206704

[hbm70242-bib-0041] Dong, X. , L. Cui , and B. W. Johnson . 2024. “Neural Mechanisms for Secondary Suppression of Emotional Distractors: Evidence From Concurrent Electroencephalography–Magnetoencephalography Data.” Emotion 24: 1907–1922. 39023970 10.1037/emo0001388

[hbm70242-bib-0042] Eastwood, J. D. , D. Smilek , and P. M. Merikle . 2003. “Negative Facial Expression Captures Attention and Disrupts Performance.” Perception & Psychophysics 65: 352–358. 12785065 10.3758/bf03194566

[hbm70242-bib-0043] Ebner, N. C. , M. Riediger , and U. Lindenberger . 2010. “Faces—A Database of Facial Expressions in Young, Middle‐Aged, and Older Women and Men: Development and Validation.” Behavior Research Methods 42: 351–362. 20160315 10.3758/BRM.42.1.351

[hbm70242-bib-0044] Eimer, M. , and A. Holmes . 2007. “Event‐Related Brain Potential Correlates of Emotional Face Processing.” Neuropsychologia 45: 15–31. 16797614 10.1016/j.neuropsychologia.2006.04.022 PMC2383989

[hbm70242-bib-0045] Engel, A. K. , and P. Fries . 2010. “Beta‐Band Oscillations—Signalling the Status Quo?” Current Opinion in Neurobiology 20: 156–165 Cognitive neuroscience. 20359884 10.1016/j.conb.2010.02.015

[hbm70242-bib-0046] Eskikurt, G. , A. D. Duru , N. Ermutlu , and Ü. İşoğlu‐Alkaç . 2024. “Evaluation of Brain Electrical Activity of Visual Working Memory With Time‐Frequency Analysis.” Clinical EEG and Neuroscience: 15500594231224014. 38225169 10.1177/15500594231224014

[hbm70242-bib-0047] Fischl, B. , M. I. Sereno , R. B. Tootell , and A. M. Dale . 1999. “High‐Resolution Intersubject Averaging and a Coordinate System for the Cortical Surface.” Human Brain Mapping 8: 272–284. 10619420 10.1002/(SICI)1097-0193(1999)8:4<272::AID-HBM10>3.0.CO;2-4 PMC6873338

[hbm70242-bib-0048] Fischl, B. , A. van der Kouwe , C. Destrieux , et al. 2004. “Automatically Parcellating the Human Cerebral Cortex.” Cerebral Cortex 14: 11–22. 14654453 10.1093/cercor/bhg087

[hbm70242-bib-0049] Fougnie, D. , and R. Marois . 2006. “Distinct Capacity Limits for Attention and Working Memory: Evidence From Attentive Tracking and Visual Working Memory Paradigms.” Psychological Science 17: 526–534. 16771804 10.1111/j.1467-9280.2006.01739.x

[hbm70242-bib-0050] Fries, P. , J. H. Reynolds , A. E. Rorie , and R. Desimone . 2001. “Modulation of Oscillatory Neuronal Synchronization by Selective Visual Attention.” Science 291: 1560–1563. 11222864 10.1126/science.1055465

[hbm70242-bib-0051] García‐Pacios, J. , D. Del Río , D. Villalobos , J. M. Ruiz‐Vargas , and F. Maestú . 2015a. “Emotional Interference‐Based Forgetting in Short‐Term Memory. Cognitive Inhibition of Pleasant but Not Unpleasant Biologically Relevant Distractors.” Frontiers in Psychology 6: 1–16. 25999894 10.3389/fpsyg.2015.00582 PMC4421942

[hbm70242-bib-0052] García‐Pacios, J. , P. Garcés , D. Del Río , and F. Maestú . 2015b. “Early Detection and Late Cognitive Control of Emotional Distraction by the Prefrontal Cortex.” Scientific Reports 5: 10046. 26067780 10.1038/srep10046 PMC4464367

[hbm70242-bib-0053] García‐Pacios, J. , P. Garcés , D. Del Río , and F. Maestú . 2017. “Tracking the Effect of Emotional Distraction in Working Memory Brain Networks: Evidence From an MEG Study.” Psychophysiology 54: 1726–1740. 28649710 10.1111/psyp.12912

[hbm70242-bib-0054] Gevins, A. , M. E. Smith , L. McEvoy , and D. Yu . 1997. “High‐Resolution EEG Mapping of Cortical Activation Related to Working Memory: Effects of Task Difficulty, Type of Processing, and Practice.” Cerebral Cortex 7: 374–385. 9177767 10.1093/cercor/7.4.374

[hbm70242-bib-0055] Gläscher, J. , M. Rose , and C. Büchel . 2007. “Independent Effects of Emotion and Working Memory Load on Visual Activation in the Lateral Occipital Complex.” Journal of Neuroscience 27: 4366–4373. 17442821 10.1523/JNEUROSCI.3310-06.2007 PMC6672316

[hbm70242-bib-0056] Gramfort, A. , M. Luessi , E. Larson , et al. 2013. “MEG and EEG Data Analysis With Mne‐Python.” Frontiers in Neuroscience 7: 267. 24431986 10.3389/fnins.2013.00267 PMC3872725

[hbm70242-bib-0057] Groppe, D. M. , T. P. Urbach , and M. Kutas . 2011. “Mass Univariate Analysis of Event‐Related Brain Potentials/Fields i: A Critical Tutorial Review.” Psychophysiology 48: 1711–1725. 21895683 10.1111/j.1469-8986.2011.01273.x PMC4060794

[hbm70242-bib-0058] Halgren, E. , T. Raij , K. Marinkovic , V. Jousmäki , and R. Hari . 2000. “Cognitive Response Profile of the Human Fusiform Face Area as Determined by MEG.” Cerebral Cortex 10: 69–81. 10639397 10.1093/cercor/10.1.69

[hbm70242-bib-0059] Hämäläinen, M. , R. Hari , R. J. Ilmoniemi , J. Knuutila , and O. V. Lounasmaa . 1993. “Magnetoencephalography—Theory, Instrumentation, and Applications to Noninvasive Studies of the Working Human Brain.” Reviews of Modern Physics 65: 413–497.

[hbm70242-bib-0060] Hart, S. G. , and L. E. Staveland . 1988. “Development of Nasa‐Tlx (Task Load Index): Results of Empirical and Theoretical Research.” In Human Mental Workload, Vol. 52 of Advances in Psychology, edited by P. A. Hancock and N. Meshkati , 139–183. North‐Holland.

[hbm70242-bib-0061] Hauk, O. 2004. “Keep It Simple: A Case for Using Classical Minimum Norm Estimation in the Analysis of EEG and MEG Data.” NeuroImage 21: 1612–1621. 15050585 10.1016/j.neuroimage.2003.12.018

[hbm70242-bib-0062] Haxby, J. V. , E. A. Hoffman , and M. I. Gobbini . 2000. “The Distributed Human Neural System for Face Perception.” Trends in Cognitive Sciences 4: 223–233. 10827445 10.1016/s1364-6613(00)01482-0

[hbm70242-bib-0063] Hinojosa, J. A. , F. Mercado , and L. Carretié . 2015. “N170 Sensitivity to Facial Expression: A Meta‐Analysis.” Neuroscience and Biobehavioral Reviews 55: 498–509. 26067902 10.1016/j.neubiorev.2015.06.002

[hbm70242-bib-0064] Hipp, J. F. , and M. Siegel . 2013. “Dissociating Neuronal Gamma‐Band Activity From Cranial and Ocular Muscle Activity in EEG.” Frontiers in Human Neuroscience 7: 338. 23847508 10.3389/fnhum.2013.00338 PMC3706727

[hbm70242-bib-0065] Horstmann, G. , O. V. Lipp , and S. I. Becker . 2012. “Of Toothy Grins and Angry Snarls–Open Mouth Displays Contribute to Efficiency Gains in Search for Emotional Faces.” Journal of Vision 12: 7. 10.1167/12.5.7 22637708

[hbm70242-bib-0066] Horstmann, G. , I. Scharlau , and U. Ansorge . 2006. “More Efficient Rejection of Happy Than of Angry Face Distractors in Visual Search.” Psychonomic Bulletin & Review 13: 1067–1073. 17484437 10.3758/bf03213927

[hbm70242-bib-0067] Houck, J. M. , and E. D. Claus . 2020. “A Comparison of Automated and Manual Co‐Registration for Magnetoencephalography.” PLoS One 15: e0232100. 32348350 10.1371/journal.pone.0232100 PMC7190172

[hbm70242-bib-0068] Iordan, A. D. , S. Dolcos , and F. Dolcos . 2013. “Neural Signatures of the Response to Emotional Distraction: A Review of Evidence From Brain Imaging Investigations.” Frontiers in Human Neuroscience 7: 200. 23761741 10.3389/fnhum.2013.00200 PMC3672684

[hbm70242-bib-0069] Jehna, M. , C. Neuper , A. Ischebeck , et al. 2011. “The Functional Correlates of Face Perception and Recognition of Emotional Facial Expressions as Evidenced by FMRI.” Brain Research 1393: 73–83. 21513918 10.1016/j.brainres.2011.04.007

[hbm70242-bib-0070] Jensen, O. , and C. D. Tesche . 2002. “Frontal Theta Activity in Humans Increases With Memory Load in a Working Memory Task.” European Journal of Neuroscience 15: 1395–1399. 11994134 10.1046/j.1460-9568.2002.01975.x

[hbm70242-bib-0071] Jokisch, D. , and O. Jensen . 2007. “Modulation of Gamma and Alpha Activity During a Working Memory Task Engaging the Dorsal or Ventral Stream.” Journal of Neuroscience 27: 3244–3251. 17376984 10.1523/JNEUROSCI.5399-06.2007 PMC6672464

[hbm70242-bib-0072] Jolly, E. 2018. “Pymer4: Connecting R and Python for Linear Mixed Modeling.” Journal of Open Source Software 3: 862.

[hbm70242-bib-0073] Kanwisher, N. , J. McDermott , and M. M. Chun . 1997. “The Fusiform Face Area: A Module in Human Extrastriate Cortex Specialized for Face Perception.” Journal of Neuroscience 17: 4302–4311. 9151747 10.1523/JNEUROSCI.17-11-04302.1997 PMC6573547

[hbm70242-bib-0074] Kim, H. 2019. “Neural Activity During Working Memory Encoding, Maintenance, and Retrieval: A Network‐Based Model and Meta‐Analysis.” Human Brain Mapping 40: 4912–4933. 31373730 10.1002/hbm.24747 PMC6865408

[hbm70242-bib-0075] Kimura, T. , and R. Matsuura . 2023. “The Content‐Dependent Effect of the n‐Back Task on Dual‐Task Performance.” Behavioural Brain Research 452: 114511. 37263422 10.1016/j.bbr.2023.114511

[hbm70242-bib-0076] Klimesch, W. , P. Sauseng , and S. Hanslmayr . 2007. “EEG Alpha Oscillations: The Inhibition‐Timing Hypothesis.” Brain Research Reviews 53: 63–88. 16887192 10.1016/j.brainresrev.2006.06.003

[hbm70242-bib-0077] Kopell, N. , M. A. Whittington , and M. A. Kramer . 2011. “Neuronal Assembly Dynamics in the beta1 Frequency Range Permits Short‐Term Memory.” Proceedings of the National Academy of Sciences of the United States of America 108: 3779–3784. 21321198 10.1073/pnas.1019676108 PMC3048142

[hbm70242-bib-0078] Lamm, C. , C. Windischberger , U. Leodolter , E. Moser , and H. Bauer . 2001. “Evidence for Premotor Cortex Activity During Dynamic Visuospatial Imagery From Single‐Trial Functional Magnetic Resonance Imaging and Event‐Related Slow Cortical Potentials.” NeuroImage 14: 268–283. 11467902 10.1006/nimg.2001.0850

[hbm70242-bib-0079] Lang, P. J. , M. M. Bradley , B. N. Cuthbert , et al. 1997. International Affective Picture System (Iaps): Technical Manual and Affective Ratings. Vol. 1, 3. NIMH Center for the Study of Emotion and Attention.

[hbm70242-bib-0080] Lavie, N. , and J. de Fockert . 2005. “The Role of Working Memory in Attentional Capture.” Psychonomic Bulletin & Review 12: 669–674. 16447380 10.3758/bf03196756

[hbm70242-bib-0081] Lavie, N. , A. Hirst , J. W. de Fockert , and E. Viding . 2004. “Load Theory of Selective Attention and Cognitive Control.” Journal of Experimental Psychology: General 133: 339–354. 15355143 10.1037/0096-3445.133.3.339

[hbm70242-bib-0082] Lee, E. , J. Kang , I. H. Park , J.‐J. Kim , and S. K. An . 2008. “Is a Neutral Face Really Evaluated as Being Emotionally Neutral?” Psychiatry Research 157: 77–85. 17804083 10.1016/j.psychres.2007.02.005

[hbm70242-bib-0083] Lee, T.‐W. , M. Girolami , and T. J. Sejnowski . 1999. “Independent Component Analysis Using an Extended Infomax Algorithm for Mixed Subgaussian and Supergaussian Sources.” Neural Computation 11: 417–441. 9950738 10.1162/089976699300016719

[hbm70242-bib-0084] Leopold, D. A. , I. V. Bondar , and M. A. Giese . 2006. “Norm‐Based Face Encoding by Single Neurons in the Monkey Inferotemporal Cortex.” Nature 442: 572–575. 16862123 10.1038/nature04951

[hbm70242-bib-0085] Leopold, D. A. , A. J. O'Toole , T. Vetter , and V. Blanz . 2001. “Prototype‐Referenced Shape Encoding Revealed by High‐Level Aftereffects.” Nature Neuroscience 4: 89–94. 11135650 10.1038/82947

[hbm70242-bib-0086] Leung, H.‐C. , D. Seelig , and J. C. Gore . 2004. “The Effect of Memory Load on Cortical Activity in the Spatial Working Memory Circuit.” Cognitive, Affective, & Behavioral Neuroscience 4: 553–563. 10.3758/cabn.4.4.553 15849897

[hbm70242-bib-0087] Li, X. , Z. Ouyang , and Y. Jia Luo . 2012. “The Cognitive Load Affects the Interaction Pattern of Emotion and Working Memory.” International Journal of Cognitive Informatics and Natural Intelligence 6: 68–81.

[hbm70242-bib-0088] Lim, S.‐L. , A. S. Bruce , and R. L. Aupperle . 2014. “The Influence of a Working Memory Task on Affective Perception of Facial Expressions.” PLoS One 9: e111074. 25347772 10.1371/journal.pone.0111074 PMC4210225

[hbm70242-bib-0089] Lin, F.‐H. , T. Witzel , S. P. Ahlfors , S. M. Stufflebeam , J. W. Belliveau , and M. S. Hämäläinen . 2006. “Assessing and Improving the Spatial Accuracy in MEG Source Localization by Depth‐Weighted Minimum‐Norm Estimates.” NeuroImage 31: 160–171. 16520063 10.1016/j.neuroimage.2005.11.054

[hbm70242-bib-0090] Mackie, M.‐A. , N. T. van Dam , and J. Fan . 2013. “Cognitive Control and Attentional Functions.” Brain and Cognition 82: 301–312. 23792472 10.1016/j.bandc.2013.05.004 PMC3722267

[hbm70242-bib-0091] MacNamara, A. , J. Schmidt , G. J. Zelinsky , and G. Hajcak . 2012. “Electrocortical and Ocular Indices of Attention to Fearful and Neutral Faces Presented Under High and Low Working Memory Load.” Biological Psychology 91: 349–356. 22951516 10.1016/j.biopsycho.2012.08.005

[hbm70242-bib-0092] Maris, E. , and R. Oostenveld . 2007. “Nonparametric Statistical Testing of EEG‐ and MEG‐Data.” Journal of Neuroscience Methods 164: 177–190. 17517438 10.1016/j.jneumeth.2007.03.024

[hbm70242-bib-0093] Michels, L. , K. Bucher , R. Lüchinger , et al. 2010. “Simultaneous EEG‐FMRI During a Working Memory Task: Modulations in Low and High Frequency Bands.” PLoS One 5: e10298. 20421978 10.1371/journal.pone.0010298 PMC2858659

[hbm70242-bib-0094] Miller, E. K. , and J. D. Cohen . 2001. “An Integrative Theory of Prefrontal Cortex Function.” Annual Review of Neuroscience 24: 167–202. 10.1146/annurev.neuro.24.1.167 11283309

[hbm70242-bib-0095] Mitchell, A. S. , R. Czajkowski , N. Zhang , K. Jeffery , and A. J. D. Nelson . 2018. “Retrosplenial Cortex and Its Role in Spatial Cognition.” Brain and Neuroscience Advances 2: 1–13. 30221204 10.1177/2398212818757098 PMC6095108

[hbm70242-bib-0096] Mitchell, D. G. V. , M. Nakic , D. Fridberg , N. Kamel , D. S. Pine , and R. J. R. Blair . 2007. “The Impact of Processing Load on Emotion.” NeuroImage 34: 1299–1309. 17161627 10.1016/j.neuroimage.2006.10.012 PMC1909754

[hbm70242-bib-0097] Monroe, J. F. , M. Griffin , A. Pinkham , et al. 2013. “The Fusiform Response to Faces: Explicit Versus Implicit Processing of Emotion.” Human Brain Mapping 34: 1–11. 21932258 10.1002/hbm.21406 PMC6870213

[hbm70242-bib-0098] Montgomery, K. J. , and J. V. Haxby . 2008. “Mirror Neuron System Differentially Activated by Facial Expressions and Social Hand Gestures: A Functional Magnetic Resonance Imaging Study.” Journal of Cognitive Neuroscience 20: 1866–1877. 18370602 10.1162/jocn.2008.20127

[hbm70242-bib-0099] Mosher, J. C. , R. M. Leahy , and P. S. Lewis . 1999. “EEG and MEG: Forward Solutions for Inverse Methods.” IEEE Transactions on Biomedical Engineering 46: 245–259. 10097460 10.1109/10.748978

[hbm70242-bib-0100] Müller‐Bardorff, M. , M. Bruchmann , M. Mothes‐Lasch , et al. 2018. “Early Brain Responses to Affective Faces: A Simultaneous EEG‐FMRI Study.” NeuroImage 178: 660–667. 29864521 10.1016/j.neuroimage.2018.05.081

[hbm70242-bib-0101] Navon, D. , and J. Miller . 2002. “Queuing or Sharing? A Critical Evaluation of the Single‐Bottleneck Notion.” Cognitive Psychology 44: 193–251. 11971632 10.1006/cogp.2001.0767

[hbm70242-bib-0102] Nolan, H. , R. Whelan , and R. B. Reilly . 2010. “Faster: Fully Automated Statistical Thresholding for EEG Artifact Rejection.” Journal of Neuroscience Methods 192: 152–162. 20654646 10.1016/j.jneumeth.2010.07.015

[hbm70242-bib-0103] Oberauer, K. 2019. “Working Memory and Attention—A Conceptual Analysis and Review.” Journal of Cognition 2: 36. 31517246 10.5334/joc.58 PMC6688548

[hbm70242-bib-0104] Ochsner, K. N. , and J. J. Gross . 2005. “The Cognitive Control of Emotion.” Trends in Cognitive Sciences 9: 242–249. 15866151 10.1016/j.tics.2005.03.010

[hbm70242-bib-0105] Olk, B. , C. Peschke , and C. C. Hilgetag . 2015. “Attention and Control of Manual Responses in Cognitive Conflict: Findings From Tms Perturbation Studies.” Neuropsychologia 74: 7–20. 25661841 10.1016/j.neuropsychologia.2015.02.008

[hbm70242-bib-0106] Pavlov, Y. G. , and B. Kotchoubey . 2022. “Oscillatory Brain Activity and Maintenance of Verbal and Visual Working Memory: A Systematic Review.” Psychophysiology 59: e13735. 33278030 10.1111/psyp.13735

[hbm70242-bib-0107] Pernet, C. , M. Latinus , T. Nichols , and G. Rousselet . 2015. “Cluster‐Based Computational Methods for Mass Univariate Analyses of Event‐Related Brain Potentials/Fields: A Simulation Study.” Journal of Neuroscience Methods 250: 85–93. 25128255 10.1016/j.jneumeth.2014.08.003 PMC4510917

[hbm70242-bib-0108] Pessoa, L. 2008. “On the Relationship Between Emotion and Cognition.” Nature Reviews Neuroscience 9: 148–158. 18209732 10.1038/nrn2317

[hbm70242-bib-0109] Pessoa, L. , S. Kastner , and L. G. Ungerleider . 2002. “Attentional Control of the Processing of Neural and Emotional Stimuli.” Cognitive Brain Research 15: 31–45. 12433381 10.1016/s0926-6410(02)00214-8

[hbm70242-bib-0110] Pessoa, L. , S. Padmala , and T. Morland . 2005. “Fate of Unattended Fearful Faces in the Amygdala Is Determined by Both Attentional Resources and Cognitive Modulation.” NeuroImage 28: 249–255. 15993624 10.1016/j.neuroimage.2005.05.048 PMC2427145

[hbm70242-bib-0111] Pitcher, D. , L. Garrido , V. Walsh , and B. C. Duchaine . 2008. “Transcranial Magnetic Stimulation Disrupts the Perception and Embodiment of Facial Expressions.” Journal of Neuroscience 28: 8929–8933. 18768686 10.1523/JNEUROSCI.1450-08.2008 PMC6670866

[hbm70242-bib-0112] Pizzagalli, D. A. , D. Lehmann , A. M. Hendrick , M. Regard , R. D. Pascual‐Marqui , and R. J. Davidson . 2002. “Affective Judgments of Faces Modulate Early Activity (Approximately 160 ms) Within the Fusiform Gyri.” NeuroImage 16: 663–677. 12169251 10.1006/nimg.2002.1126

[hbm70242-bib-0113] Posamentier, M. T. , and H. Abdi . 2003. “Processing Faces and Facial Expressions.” Neuropsychology Review 13: 113–143. 14584908 10.1023/a:1025519712569

[hbm70242-bib-0114] Proskovec, A. L. , E. Heinrichs‐Graham , and T. W. Wilson . 2019. “Load Modulates the Alpha and Beta Oscillatory Dynamics Serving Verbal Working Memory.” NeuroImage 184: 256–265. 30213775 10.1016/j.neuroimage.2018.09.022 PMC6230485

[hbm70242-bib-0115] Proskovec, A. L. , A. I. Wiesman , E. Heinrichs‐Graham , and T. W. Wilson . 2019. “Load Effects on Spatial Working Memory Performance Are Linked to Distributed Alpha and Beta Oscillations.” Human Brain Mapping 40: 3682–3689. 31077487 10.1002/hbm.24625 PMC6865445

[hbm70242-bib-0116] Rellecke, J. , W. Sommer , and A. Schacht . 2012. “Does Processing of Emotional Facial Expressions Depend on Intention? Time‐Resolved Evidence From Event‐Related Brain Potentials.” Biological Psychology 90: 23–32. 22361274 10.1016/j.biopsycho.2012.02.002

[hbm70242-bib-0117] Roesch, E. B. , D. Sander , C. Mumenthaler , D. Kerzel , and K. R. Scherer . 2010. “Psychophysics of Emotion: The Quest for Emotional Attention.” Journal of Vision 10: 4–9. 10.1167/10.3.4 20377281

[hbm70242-bib-0118] Rottschy, C. , R. Langner , I. Dogan , et al. 2012. “Modelling Neural Correlates of Working Memory: A Coordinate‐Based Meta‐Analysis.” NeuroImage 60: 830–846. 22178808 10.1016/j.neuroimage.2011.11.050 PMC3288533

[hbm70242-bib-0119] Said, C. P. , J. V. Haxby , and A. Todorov . 2011. “Brain Systems for Assessing the Affective Value of Faces.” Philosophical Transactions of the Royal Society, B: Biological Sciences 366: 1660–1670. 10.1098/rstb.2010.0351 PMC3130375 21536552

[hbm70242-bib-0120] Salazar, R. F. , N. M. Dotson , S. L. Bressler , and C. M. Gray . 2012. “Content‐Specific Fronto‐Parietal Synchronization During Visual Working Memory.” Science 338: 1097–1100. 23118014 10.1126/science.1224000 PMC4038369

[hbm70242-bib-0121] Scharinger, C. , A. Soutschek , T. Schubert , and P. Gerjets . 2015. “When Flanker Meets the n‐Back: What EEG and Pupil Dilation Data Reveal About the Interplay Between the Two Central‐Executive Working Memory Functions Inhibition and Updating.” Psychophysiology 52: 1293–1304. 26238380 10.1111/psyp.12500

[hbm70242-bib-0122] Scharinger, C. , A. Soutschek , T. Schubert , and P. Gerjets . 2017. “Comparison of the Working Memory Load in n‐Back and Working Memory Span Tasks by Means of EEG Frequency Band Power and p300 Amplitude.” Frontiers in Human Neuroscience 11: 6. 28179880 10.3389/fnhum.2017.00006 PMC5263141

[hbm70242-bib-0123] Schindler, S. , and F. Bublatzky . 2020. “Attention and Emotion: An Integrative Review of Emotional Face Processing as a Function of Attention.” Cortex 130: 362–386. 32745728 10.1016/j.cortex.2020.06.010

[hbm70242-bib-0124] Schmiedek, F. , M. Lövdén , and U. Lindenberger . 2014. “A Task Is a Task Is a Task: Putting Complex Span, n‐Back, and Other Working Memory Indicators in Psychometric Context.” Frontiers in Psychology 5: 1475. 25566149 10.3389/fpsyg.2014.01475 PMC4274887

[hbm70242-bib-0125] Schroeder, S. C. Y. , F. Ball , and N. A. Busch . 2018. “The Role of Alpha Oscillations in Distractor Inhibition During Memory Retention.” European Journal of Neuroscience 48: 2516–2526. 29381823 10.1111/ejn.13852

[hbm70242-bib-0126] Schupp, H. T. , T. Flaisch , J. Stockburger , and M. Junghöfer . 2006. “Emotion and Attention: Event‐Related Brain Potential Studies.” Progress in Brain Research 156: 31–51. 17015073 10.1016/S0079-6123(06)56002-9

[hbm70242-bib-0127] Schweizer, S. , A. B. Satpute , S. Atzil , et al. 2019. “The Impact of Affective Information on Working Memory: A Pair of Meta‐Analytic Reviews of Behavioral and Neuroimaging Evidence.” Psychological Bulletin 145: 566–609. 31021136 10.1037/bul0000193 PMC6526745

[hbm70242-bib-0128] Ségonne, F. , A. M. Dale , E. Busa , et al. 2004. “A Hybrid Approach to the Skull Stripping Problem in Mri.” NeuroImage 22: 1060–1075. 15219578 10.1016/j.neuroimage.2004.03.032

[hbm70242-bib-0129] Skaramagkas, V. , G. Giannakakis , E. Ktistakis , et al. 2021. “Review of Eye Tracking Metrics Involved in Emotional and Cognitive Processes.” IEEE Reviews in Biomedical Engineering 16: 260–277. 10.1109/RBME.2021.3066072 33729950

[hbm70242-bib-0130] Skinner, A. L. , and C. P. Benton . 2010. “Anti‐Expression Aftereffects Reveal Prototype‐Referenced Coding of Facial Expressions.” Psychological Science 21: 1248–1253. 20713632 10.1177/0956797610380702

[hbm70242-bib-0131] Spiering, L. , and O. Dimigen . 2024. “(Micro)saccade‐Related Potentials During Face Recognition: A Study Combining EEG, Eye‐Tracking, and Deconvolution Modeling.” Attention, Perception, & Psychophysics 87, no. 1: 1–22. 10.3758/s13414-024-02846-1 PMC11845548 38296873

[hbm70242-bib-0132] Srinivasan, R. , W. R. Winter , and P. L. Nunez . 2006. “Source Analysis of EEG Oscillations Using High‐Resolution EEG and MEG.” In Event‐Related Dynamics of Brain Oscillations, edited by C. Neuper and W. Klimesch , 29–42. Elsevier.vol. 159 of Progress in Brain Research. 10.1016/S0079-6123(06)59003-X PMC1995013 17071222

[hbm70242-bib-0133] Taulu, S. , and J. Simola . 2006. “Spatiotemporal Signal Space Separation Method for Rejecting Nearby Interference in MEG Measurements.” Physics in Medicine and Biology 51: 1759–1768. 16552102 10.1088/0031-9155/51/7/008

[hbm70242-bib-0134] Taulu, S. , J. Simola , and M. Kajola . 2005. “Applications of the Signal Space Separation Method.” IEEE Transactions on Signal Processing 53: 3359–3372.

[hbm70242-bib-0135] Tavares, T. P. , K. Logie , and D. G. Mitchell . 2016. “Opposing Effects of Perceptual Versus Working Memory Load on Emotional Distraction.” Experimental Brain Research 234: 2945–2956. 27329606 10.1007/s00221-016-4697-2

[hbm70242-bib-0136] Tomasi, D. , L. Chang , E. C. Caparelli , and T. Ernst . 2007. “Different Activation Patterns for Working Memory Load and Visual Attention Load.” Brain Research 1132: 158–165. 17169343 10.1016/j.brainres.2006.11.030 PMC1831676

[hbm70242-bib-0137] Tombu, M. , and P. Jolicoeur . 2003. “A Central Capacity Sharing Model of Dual‐Task Performance.” Journal of Experimental Psychology: Human Perception and Performance 29: 3–18. 12669744 10.1037//0096-1523.29.1.3

[hbm70242-bib-0138] Unni, A. , K. Ihme , M. Jipp , and J. W. Rieger . 2017. “Assessing the Driver's Current Level of Working Memory Load With High Density Functional Near‐Infrared Spectroscopy: A Realistic Driving Simulator Study.” Frontiers in Human Neuroscience 11: 167. 28424602 10.3389/fnhum.2017.00167 PMC5380755

[hbm70242-bib-0139] van Dillen, L. F. , and B. Derks . 2012. “Working Memory Load Reduces Facilitated Processing of Threatening Faces: An ERP Study.” Emotion 12: 1340–1349. 22642340 10.1037/a0028624

[hbm70242-bib-0140] Van Dillen, L. F. , and S. L. Koole . 2009. “How Automatic Is “Automatic Vigilance”? The Role of Working Memory in Attentional Interference of Negative Information.” Cognition and Emotion 23: 1106–1117.

[hbm70242-bib-0141] Vann, S. D. , J. P. Aggleton , and E. A. Maguire . 2009. “What Does the Retrosplenial Cortex Do?” Nature Reviews Neuroscience 10: 792–802. 19812579 10.1038/nrn2733

[hbm70242-bib-0142] von Lühmann, A. , Z. Boukouvalas , K.‐R. Müller , and T. Adal . 2019. “A New Blind Source Separation Framework for Signal Analysis and Artifact Rejection in Functional Near‐Infrared Spectroscopy.” NeuroImage 200: 72–88. 31203024 10.1016/j.neuroimage.2019.06.021

[hbm70242-bib-0143] Vuilleumier, P. 2005. “How Brains Beware: Neural Mechanisms of Emotional Attention.” Trends in Cognitive Sciences 9: 585–594. 16289871 10.1016/j.tics.2005.10.011

[hbm70242-bib-0144] Vuilleumier, P. , J. Armony , R. Dolan , et al. 2003. “Reciprocal Links Between Emotion and Attention.” Human Brain Function 2: 419–444.

[hbm70242-bib-0145] Vuilleumier, P. , and Y.‐M. Huang . 2009. “Emotional Attention: Uncovering the Mechanisms of Affective Biases in Perception.” Current Directions in Psychological Science 18: 148–152.

[hbm70242-bib-0146] Vuilleumier, P. , and G. Pourtois . 2007. “Distributed and Interactive Brain Mechanisms During Emotion Face Perception: Evidence From Functional Neuroimaging.” Neuropsychologia 45: 174–194. 16854439 10.1016/j.neuropsychologia.2006.06.003

[hbm70242-bib-0147] Wang, L. , C. Feng , X. Mai , et al. 2016. “The Impact of Perceptual Load on the Non‐Conscious Processing of Fearful Faces.” PLoS One 11: e0154914. 27149273 10.1371/journal.pone.0154914 PMC4858266

[hbm70242-bib-0148] Watanabe, K. , and S. Funahashi . 2014. “Neural Mechanisms of Dual‐Task Interference and Cognitive Capacity Limitation in the Prefrontal Cortex.” Nature Neuroscience 17: 601–611. 24584049 10.1038/nn.3667

[hbm70242-bib-0149] Weidner, E. M. , S. Moratti , S. Schindler , P. Grewe , C. G. Bien , and J. Kissler . 2024. “Amygdala and Cortical Gamma‐Band Responses to Emotional Faces Are Modulated by Attention to Valence.” Psychophysiology 61: e14512. 38174584 10.1111/psyp.14512

[hbm70242-bib-0150] Whalen, C. , E. L. Maclin , M. Fabiani , and G. Gratton . 2008. “Validation of a Method for Coregistering Scalp Recording Locations With 3d Structural MR Images.” Human Brain Mapping 29: 1288–1301. 17894391 10.1002/hbm.20465 PMC6871211

[hbm70242-bib-0151] Wickens, C. D. 2014. “The Structure of Attentional Resources.” In Attention and Performance VIII, 239–257. Psychology Press.

[hbm70242-bib-0152] Winston, J. S. , J. O'Doherty , and R. J. Dolan . 2003. “Common and Distinct Neural Responses During Direct and Incidental Processing of Multiple Facial Emotions.” NeuroImage 20: 84–97. 14527572 10.1016/s1053-8119(03)00303-3

[hbm70242-bib-0153] Xu, Q. , C. Ye , S. Gu , et al. 2021. “Negative and Positive Bias for Emotional Faces: Evidence From the Attention and Working Memory Paradigms.” Neural Plasticity 2021: 8851066. 34135956 10.1155/2021/8851066 PMC8178010

[hbm70242-bib-0154] Yang, P. , M. Wang , Z. Jin , and L. Li . 2015. “Visual Short‐Term Memory Load Modulates the Early Attention and Perception of Task‐Irrelevant Emotional Faces.” Frontiers in Human Neuroscience 9: 490. 26388763 10.3389/fnhum.2015.00490 PMC4558536

[hbm70242-bib-0155] Yoon, J. H. , C. E. Curtis , and M. D'Esposito . 2006. “Differential Effects of Distraction During Working Memory on Delay‐Period Activity in the Prefrontal Cortex and the Visual Association Cortex.” NeuroImage 29: 1117–1126. 16226895 10.1016/j.neuroimage.2005.08.024

