# Supplementary material for: Neurophysiological Basis of Emotional Face Perception and Working Memory Load in a Dual‐Task MEG Study
Source: Hum Brain Mapp. 2025 Jun 9;46(8):e70242. doi: 10.1002/hbm.70242 (PMC12147946; doi:10.1002/hbm.70242)
Supplement: Supplementary file 1 — Data S1 Supporting Information. [file HBM-46-e70242-s001.pdf]

Supporting Information

A | SUPPLEMENTARY MATERIAL

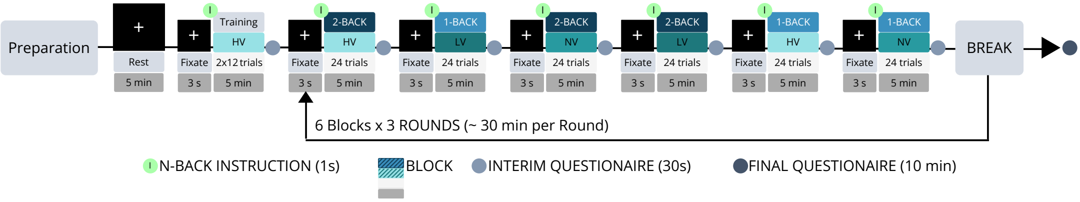

FIGURE 1 Overview of the experiment procedure. The order of the conditions is an illustrative example. The provided times are approximated. Conditions were presented randomly within one round without repetition. LV: low valence; NV: neutral valence; HV: high valence; I: instruction.

TABLE 1 Summary of the behavioural linear mixed-effects models analyzing the main effects emotional expression and working memory (WM) load as well as their interaction during the facial emotion discrimination and n-back encoding/retrieval phase.

**Measure**	**Effect**	***df1***	***df2***	***F***	***p***
**Performance**					
	Workload	230	1	0.05	0.826
Errors Facial Emotion Discrimination	Emotion	230	2	24.52	<0.001***
	Workload * Emotion	230	2	0.30	0.741
	Workload	230	1	68.27	<0.001***
Errors N-back Retrieval	Emotion	230	2	0.50	0.607
	Workload * Emotion	230	2	0.01	0.988
	Workload	230	1	9.91	0.002**
Reaction Time Facial Emotion Discrimination	Emotion	230	2	15.27	<0.001***
	Workload * Emotion	230	2	0.11	0.898
	Workload	230	1	15.21	<0.001***
Reaction Time N-back Retrieval	Emotion	230	2	2.15	0.118
	Workload * Emotion	230	2	0.28	0.754
**Subjective Ratings**					
	Workload	230	1	6.27	0.013*
Valence (SAM Subscale)	Emotion	230	2	18.80	<0.001***
	Workload * Emotion	230	2	0.99	0.373
	Workload	230	1	41.08	<0.001***
Arousal (SAM Subscale)	Emotion	230	2	3.33	0.038*
	Workload * Emotion	230	2	1.75	0.175
	Workload	230	1	75.71	<0.001***
Effort (NASA TLX Subscale)	Emotion	230	2	1.93	0.148
	Workload * Emotion	230	2	0.93	0.396

Note. Significance level: * indicates $p < 0.05$, ** indicates $p < 0.01$, and *** indicates $p < 0.001$.

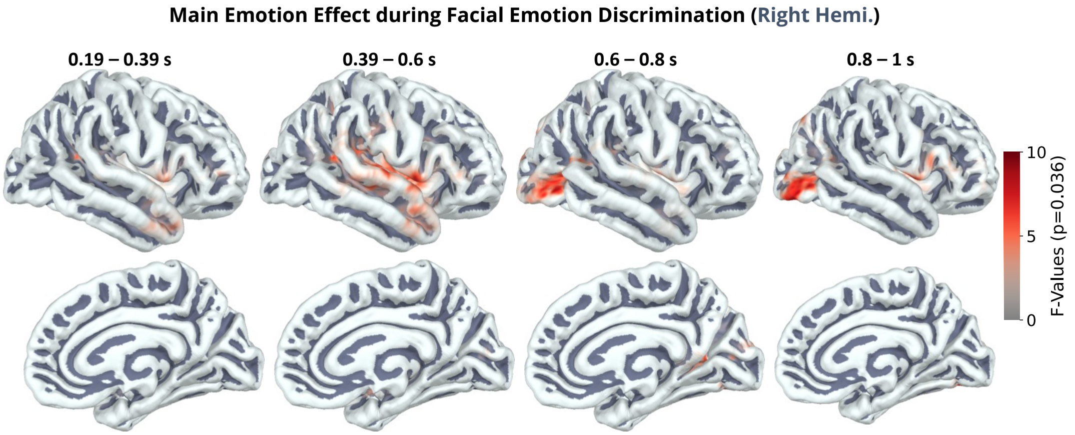

FIGURE 2 Spatiotemporal *F*-statistic cluster of the main effect emotional expression and the temporally evolving spatial distribution of modulated event-related magnetic fields (ERFs).

A.1 | Exploratory Correlation Analyses within and across Spatiotemporal Event-related Magnetic Fields Clusters

The spatiotemporal clustering of event-related magnetic fields (ERFs) does not permit inferring potential relationships between cluster regions and time intervals on a trial-by-trial level because it is based on averaged MEG activations across trials. This folalow-up analysis focuses on relations within and between regions and time intervals involved in the process of facial emotion discrimination as revealed by the ERF sub-clusters. The objective was to identify the processing stream across time intervals and ROIs for each condition.

For this purpose, we refined the spatiotemporal ERF clusters by taking only t-values corresponding to a significance threshold of $\alpha < 0.01$ ($t > 2.715$ and $t < -2.715$). Each cluster was segmented into four time intervals and significant t-values within each time interval were average over time. Next, we extracted two spatially separated regions of interest (ROIs) maximally distinguishing the conditions of the comparison, as determined by their t-value. To obtain the primary peak (ROI 1), we drew a 10 mm radius around the maximum. To obtain the secondary peak (ROI 2), we applied the constraint that it must be located at least 20 mm from the primary peak. Thus, the secondary peak is the maximum when excluding all points in a 20 mm radius around and within the primary peak. The first rule ensures that the selected ROI captures a sufficient number of vertices, while still being focused on a functionally meaningful region. The second 20 mm separation rule between the primary and secondary peaks ensures that the two regions are spatially distinct. This is important to prevent artificial correlation that may arise due to point-spread function effects.

The ROIs per post-hoc comparison, cluster, and binned time interval from the significant ERF clusters (i.e., from each panel in Figure 4 in the main text) are depicted in the Supplementary Figure 3. MNI coordinates of the ROIs (i.e., peaks per bin), size of the defined ROI (in vertices), and associated anatomical labels based on the Automated Anatomical Labelling (AAL) atlas and Brodmann atlas (BA) are provided in the Supplementary Table 2.

The source-space transformation was performed using the same approach as for the time-locked ERF and oscillatory power analyses. The Minimum Norm Estimate (MNE) was applied subject-wise to epoched data as inverse method, using an SNR value of 1 (Gramfort et al., 2013; Lin et al., 2006). We extracted the individual source activity,

TABLE 2 Descriptive parameters of the regions of interest (ROIs) for each comparison, cluster per hemisphere, and time interval of interest (TOI). Labels were defined with the aid of the Automated Anatomical Labelling (AAL) atlas and Brodmann atlas.

Comparison	Cluster	Hemi.	TOI	Primary Peak				Secondary Peak			
				MNI Coordinates	Vertices in ROI	Label	BA	MNI Coordinates	Vertices in ROI	Label	BA
LV - NV	1	left	1	[-26, -92, 8]	38	OFA L	18	[-26, -93, -15]	7	Lingual L	18
			2	[-29, -92, -8]	38	OFA L	18	[-58, -17, 1]	15	Mid Temporal L	22
			3	[-35, -82, -14]	33	FFA L	19	[-24, -89, 4]	7	OFA L	18
			4	[-39, -85, -9]	38	OFA L	19	[-13, -97, -12]	12	Lingual L	18
		right	1	[29, -92, -8]	33	OFA R	18	[19, -81, -12]	8	Lingual R	18
			2	[55, 4, 3]	41	FO R	44	[39, -24, 0]	11	STS R	41
			3	[52, 0, 5]	39	FO R	44	[51, 30, 4]	16	IFG R	45
			4	[54, 2, 3]	40	FO R	44	[38, 0, 2]	10	Insula R	13
LV - HV	1	left	1	[-23, -98, 0]	36	OFA L	18	[-42, -80, -10]	8	OFA L	19
			2	[-38, -79, -13]	40	FFA L	19	[-29, -89, 13]	13	OFA L	19
			3	[-39, -85, -9]	38	OFA L	19	[-50, -20, 6]	10	STS L	41
			4	[-41, -85, 0]	41	OFA L	18	[-27, -60, 43]	22	Sup Parietal L	7
		right	1	[23, -94, -11]	34	OFA R	18	[57, -1, -26]	10	Mid Temporal R	21
			2	[57, -1, -26]	32	Mid Temporal R	21	[33, -88, -1]	7	OFA R	18
			3	[44, -76, -9]	37	OFA R	19	[53, -56, 10]	17	Mid Temporal R	37
			4	[41, -78, -13]	37	OFA R	19	[52, 3, 3]	11	FO R	44
	2	left	1	[-22, 30, 32]	45	Sup Frontal L	8	[-6, 3, 65]	10	SMA L	6
			2	[-41, 35, 22]	54	IFG L	46	[-14, 14, 57]	9	Sup Frontal L	6
			3	[-11, 28, 50]	30	Sup Frontal L	8	[-36, 47, 18]	10	Mid Frontal L	10
			4	[-15, 8, 60]	35	Sup Frontal L	6	[-29, 38, 19]	7	Mid Frontal L	10

Note. MNI: Montreal Neurological Institute space; BA: Brodmann area; L: left; R: right; LV: low valence; NV: neutral valence; HV: high valence; OFA: occipital face area; FFA: fusiform face area; Mid: middle; Sup: superior; FO: frontal operculum; SMA: supplementary motor area. LV-NV Clusters and LV-HV Cluster 1 with TOIs ranging from (1) 190 - 389 ms, (2) 390 - 600 ms, (3) 600 - 800 ms, (4) 800 - 1,000 ms. LV-HV Cluster 2 with TOIs ranging from (1) 400 - 550 ms, (2) 550 - 700 ms, (3) 700 - 850 ms and (4) 850 to 1,000 ms.

which had been previously transformed into a common space, from the specified ROIs for each cluster, time interval, and condition. Source estimates per cluster, time interval, and ROI were correlated subject-wise (as compared to the group-level approach in the cluster analysis) using Spearman rank correlations (r_s). This allowed quantifying the spatiotemporal relationships within and between time intervals and clusters across trials in each subject.

To discern significant relationships between time intervals and clusters across conditions, we calculated the t-value of each correlation coefficient and computed the grand average of the t-values across participants per condition and correlation coefficient. Correlations with a mean t-value above the α-threshold corresponding to a Bonferroni-corrected p-value of 0.05 were defined as statistically significant relationships. The significance threshold was $-3.91 < t > 3.91$ for the correlation coefficients of the clusters LV – NV (LV: low valence; NV: neutral valence) and $-4.19 < t > 4.19$ for those of clusters LV – HV (HV: high valence).

Our results revealed that significant correlations occurred mainly between temporally and spatially close ROIs (Supplementary Figure 3). All correlation matrices are provided in the Supplementary Figure 4. For all conditions, we observed strong correlations between ROIs located in face-specific occipital regions, that are the OFA, and FFA, as well as lingual regions within the same and across adjacent time intervals.

Using the ROIs extracted from the negative-neutral expression contrast (Figure 4A, B), neutral faces elicited additional correlation between OFA activation in early time intervals (390 – 600 ms) to later OFA activation (600 – 800 ms, r_s= .56; and 800 – 1000 ms, r_s= .55) and FFA activation (600 – 800 ms, r_s= .56; Supplementary Figure 3A). Fur-

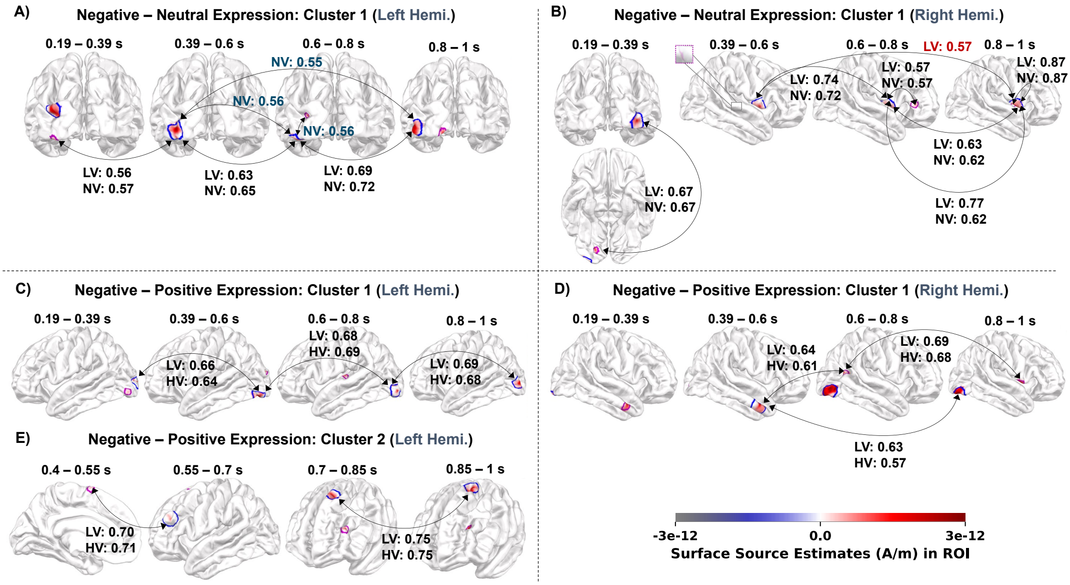

FIGURE 3 Regions of interest (ROIs) comprising the primary peak (blue outline) and secondary peak (magenta outline) per comparison, cluster, and time interval (see Figure 4). A) Caudal perspective for the left hemisphere and B) caudal and inferior (180 - 390 ms) as well as lateral perspective (390 - 1000 ms) for the right hemisphere of the contrast negative - neutral emotional expressions (Figure 4A, B). Lateral perspective for C) the left and D) right hemisphere as well as E) medial (400 - 550 ms), lateral (550 - 700 ms), and frontal (700 - 1000 ms) perspective for the second left hemispheric cluster of the contrast negative - positive emotional expressions (Figure 4C-E). Significant spatiotemporal correlation coefficients within and among the ROIs and time intervals are labelled.

thermore, we observed a strong correlation of activation in the right FO across time intervals (390 – 600 ms to 600 – 800 ms, NV: r_s= .72, LV: r_s= .74), co-activation of the right FO (600 – 800 ms) and right IFG within the same time interval (600 – 800 ms, NV and LV: r_s= .57; Supplementary Figure 3B). Moreover, the right FO was correlated with insular activation across time intervals (600 – 800 ms to 800 - 1000 ms, NV: r_s= .62, LV: r_s= .63) and within the final time interval (800 - 1000 ms, NV and LV: r_s= .87). For negative faces, we observed an additional correlation between early FO engagement (390 – 600 ms) and later insula activation (800 – 1000 ms) in the right hemisphere (r_s= .57; Supplementary Figure 3B).

Using the ROIs of the negative-positive expression contrast (Figure 4C-E), activation of the right mid-temporal sulcus (600 – 800 ms) was correlated with the right FO (800 – 1000 ms, HV: r_s= .68, LV: r_s= .69; Supplementary Figure 3D). Moreover, there were significant correlations in the second negative-positive expression cluster between the left supplementary motor area (SMA; 400 – 550 ms) and left IFG (550 – 700 ms, HV: r_s= .71, LV: r_s= .70) as well as within the superior frontal lobe in neighbouring time intervals (700 – 850 to 850 – 1000 ms, HV: r_s= .75, LV: r_s= .75; Supplementary Figure 3E).

The correlation analysis demonstrated that the processing stream for faces has few temporally and spatially distant connections within the spatiotemporal ERF clusters. Most connections were observed in face-processing-related occipital regions, the superior temporal sulcus, the insula, and inferior frontal regions at the same or adjacent time intervals. Since a correlational approach with few assumptions and no definition of direction was chosen, the possibility of moderators and other sources of activity cannot be excluded.

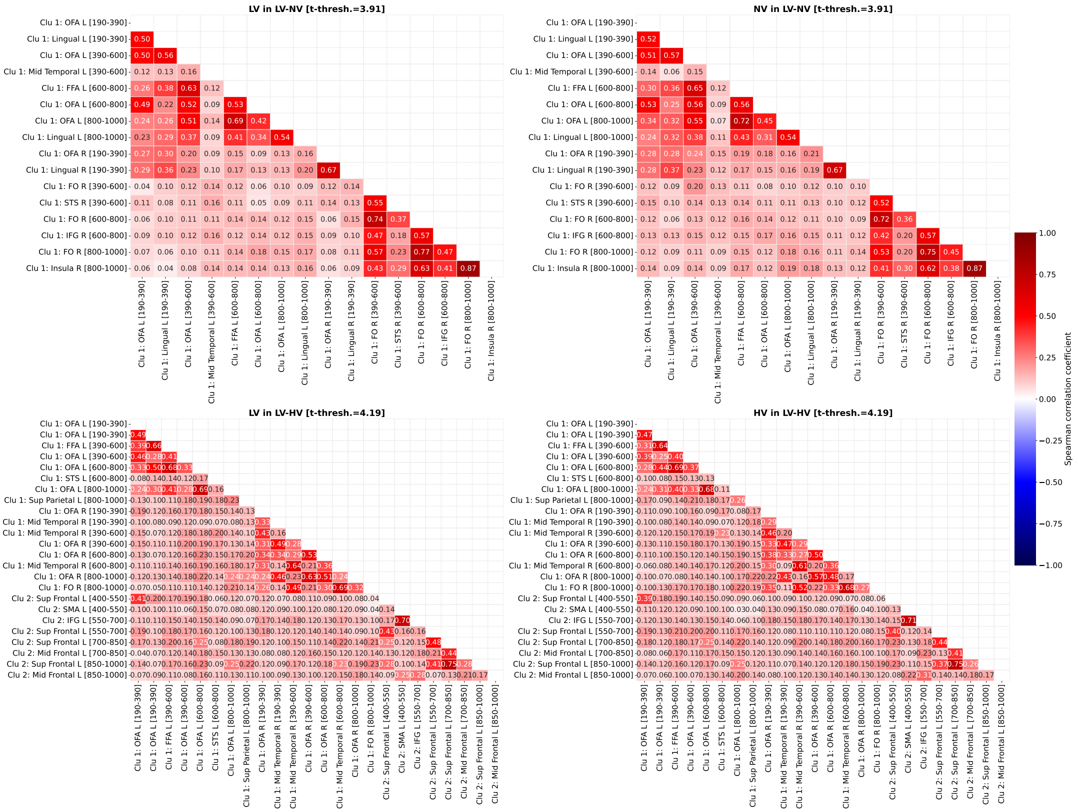

FIGURE 4 Correlation matrices of the trial-by-trial Spearman rank correlations between regions of interest (ROIs). ROIs were extracted from significant spatiotemporal event-related magnetic fields (ERFs) for each comparison, cluster per hemisphere, and time interval of interest (TOI; Figure 4). Labels were defined using the Automated Anatomical Labeling (AAL) atlas and Brodmann atlas. Clu: Cluster; L: left; R: right; LV: low valence; NV: neutral valence; HV: high valence; OFA: occipital face area; FFA: fusiform face area; Mid: middle; Sup: superior; FO: frontal operculum; SMA: supplementary motor area. LV-NV clusters and LV-HV Cluster 1 with TOIs ranging from (1) 190–389 ms, (2) 390–600 ms, (3) 600–800 ms, and (4) 800–1,000 ms. LV-HV Cluster 2 with TOIs ranging from (1) 400–550 ms, (2) 550–700 ms, (3) 700–850 ms, and (4) 850–1,000 ms.

A.2 | Exploratory Correlation Analyses of Evoked Oscillatory Power and Memory Performance

We investigated the role of working memory (WM) load-driven modulations in the alpha and beta band cluster and their relationship with high WM load performance (Bonnefond and Jensen, 2012; Proskovec et al., 2019). This was done by correlating individual cluster-based alpha- and low beta-band power modulations (directional change $HW - LW$) with performance measures (reaction time and errors) in the high WM load n-back scenario. Periodic power represents putative oscillations associated with physiological, cognitive, and behavioural states (Donoghue et al., 2020). To extract periodic power, the aperiodic ($1/f$ like) component was removed from the power spectral density, averaged across epochs, for each participant, condition, and vertex. First, the $1/f$ decay was estimated without the knee parameter using the fooof algorithm (v. 1.1.0; Donoghue et al., 2020). Then, the $1/f$ component was subtracted from the original power spectral density (both in linear space without log-transformation; Gyurkovics et al., 2021). The resulting periodic power was then averaged across band frequencies and vertices within the significant cluster. Spearman rank correlations (r_s) were computed, with z-standardised variables.

The analysis revealed a positive relationship between periodic power modulation in the alpha band cluster and retrieval reaction time during the high WM load n-back subtask (Supplementary Table 3 and Supplementary Figure 5). There was a significant correlation between directional oscillatory changes in the alpha band cluster ($HW - LW$) and retrieval reaction time ($r_s = .375$, $p = .02$). No relationship was observed between alpha band modulation and retrieval accuracy, nor between beta band power modulation and WM task performance. Our results suggest that temporo-occipital alpha suppression (i.e., a decrease in alpha band power within the cluster) under high WM load serves as a compensatory mechanism to maintain task performance by enhancing attention allocation and sensory processing. However, this alpha band modulation does not correlate with n-back retrieval accuracy under high WM load (Supplementary Table 3), indicating that it is not directly involved in maintaining the to-be-retrieved information in WM. Importantly, the relationship between oscillatory modulation and n-back retrieval time was restricted to the cluster regions (global alpha power $HW - LW$ x HW reaction time: $r_s = .235$, $p = .155$).

To conclude, WM load-driven modulation in the alpha band cluster likely represents a compensatory mechanism to enhance attention allocation and information processing under high WM load.

TABLE 3 Correlation analyses between directional modulation ($HW - LW$) in individual periodic alpha and low beta cluster power and n-back performance during high WM load (reaction time and errors).

	**Reaction Time in HW**		**Errors in HW**	
**Frequency Band Modulation**	$r_{spearman}$	***p***	$r_{spearman}$	***p***
Alpha $HW - LW$	.375	.020*	-.032	.850
Low Beta $HW - LW$	.118	.482	-.095	.570

Note. HW: High WM load; LW: Low WM load; $HW - LW$: directional change; $r_{spearman}$: Spearman rank correlation; Significance level: * indicates $p < 0.05$, ** indicates $p < 0.01$, and *** indicates $p < 0.001$.

TABLE 4 Summary of the gaze-related linear mixed-effects models analysing the main effects emotional expression and WM load as well as their interaction during the facial emotion discrimination and n-back encoding/retrieval phase.

Gaze-related Measure	Effect	*df1*	*df2*	*F*	*p*
**Facial Emotion Discrimination**					
	Workload	195	1	0.12	0.731
Mean Fixation Duration on Face	Emotion	195	2	4.10	0.018
	Workload * Emotion	195	2	1.19	0.305
	Workload	195	1	2.87	0.092
Count of Fixations on Face	Emotion	195	2	8.17	<0.001***
	Workload * Emotion	195	2	0.90	0.409
	Workload	195	1	78.99	<0.001***
Mean Pupil Dilation in Fixations	Emotion	195	2	1.76	0.176
	Workload * Emotion	195	2	4.46	0.013
**Visuo-spatial Working Memory**					
	Workload	195	1	7.10	0.008
Mean Fixation Duration on Face	Emotion	195	2	0.53	0.590
	Workload * Emotion	195	2	0.11	0.895
	Workload	195	1	0.43	0.513
Count of Fixations on Face	Emotion	195	2	1.81	0.167
	Workload * Emotion	195	2	0.87	0.419
	Workload	195	1	16.65	<0.001***
Mean Pupil Dilation in Fixations	Emotion	195	2	1.95	0.145
	Workload * Emotion	195	2	1.81	0.167
	Workload	176.25	1	13.26	<0.001***
Onset of Fixation on Target Position Square	Emotion	177.21	2	1.65	0.195
	Workload * Emotion	176.22	2	0.34	0.714
	Workload	185.38	1	2.32	0.129
Onset of Fixation on Target Colour Square	Emotion	184.24	2	0.21	0.812
	Workload * Emotion	184.26	2	1.03	0.359
	Workload	195	1	0.08	0.777
Count of Fixation on Target Position Square	Emotion	195	2	1.06	0.347
	Workload * Emotion	195	2	4.35	0.014
	Workload	195	1	0.06	0.814
Count of Fixation on Target Colour Square	Emotion	195	2	0.69	0.502
	Workload * Emotion	195	2	3.36	0.037

Note. Significance level: * indicates $p < 0.05$, ** indicates $p < 0.01$, and *** indicates $p < 0.001$.

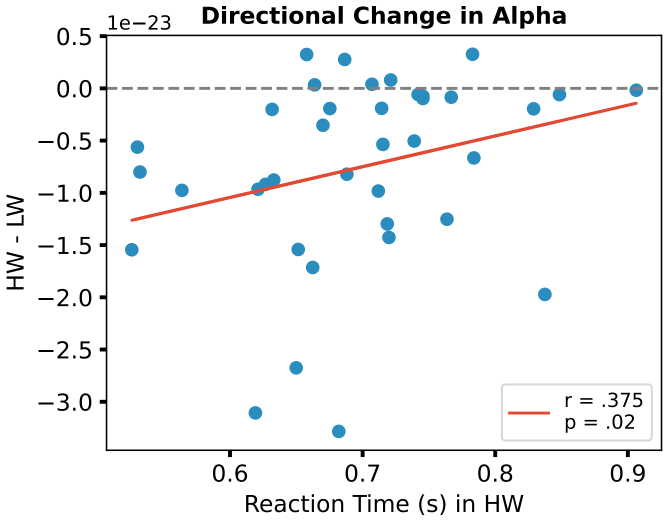

FIGURE 5 Correlation between individual periodic working memory (WM) load-based alpha band modulation (i.e., directional oscillatory changes $HW - LW$) and n-back retrieval reaction time under high WM load. The analysis focuses on individual periodic alpha band power, averaged across cluster vertices identified through a permutation-based cluster analysis. HW: High WM load; LW: Low WM load; r: Spearman rank correlation.

A.3 | Effects of Dual-Task Phase on Gaze Behaviour

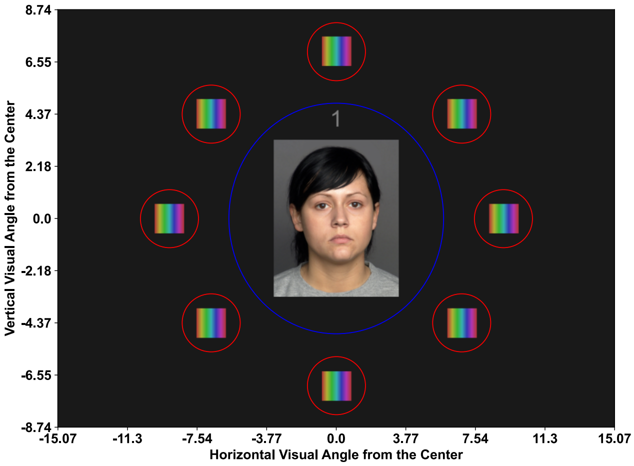

FIGURE 6 Defined regions of interest (ROIs) around the face stimuli (blue circle) and squares (red circles) for the fixation-related eye-tracking analysis.

A.3.1 | Fixations on Face Regions

To analyse whether one half of the face was preferred during fixations depending on the dual-task phase, we vertically divided the face ROI into an upper part, including the eyes, and a lower part, including the mouth. The relative fixation count and duration were extracted subject-wise for each emotional expression, facial region, and dual-task phase (i.e., during the emotion discrimination; ED; and n-back encoding/retrieval phase; N-back). The three-way ANOVA with the factors emotional expression (HV, NV, LV) x facial region (upper, lower) x task phase (ED, N-back) revealed a significant main effect of face region on both relative fixation count ($F_{(1,468)} = 243.27$, $p < .001$) and duration ($F_{(1,429)} = 252.76$, $p <$ .001). There was no main effect of emotional expression nor a significant interaction with emotional expression. Task phase had a significant main effect on relative fixation count ($F_{(1,468)} = 71.03$, $p < .001$) and duration ($F_{(1,429)} = 140.49$, $p < .001$).

Importantly, we found a significant interaction between face region and task phase, affecting both relative fixation count ($F_{(1,468)} = 206.22$, $p < .001$) and duration ($F_{(1,429)} = 202.66$, $p < .001$; Supplementary Figure 7). The interaction indicated that the preference for a facial region depended on the specific dual-task phase and subtask. The lower face region was favoured in fixations only during emotion discrimination (ED lower-upper ROI relative fixation count: M = 0.482%; 95%*CI* [0.315, 0.627]; relative fixation duration: M = 0.386%; 95%*CI* [0.259, 0.503]), but not during the n-back encoding/retrieval phase (N-back lower-upper ROI relative fixation count: M = 0.019%; 95%*CI* [-0.070,

0.103]; relative fixation duration: M = 0.021%; 95%CI [-0.037, 0.080]; Supplementary Figure 7). When comparing task phase effects per region, more fixations were positioned in the lower face region during the emotion discrimination compared to the n-back encoding/retrieval phase (ED-N-back lower ROI: M = 0.367%; 95%CI [0.306, 0.426]) and significantly fewer fixations were located in the upper face region during emotion discrimination (ED-N-back upper ROI: M = -0.095%; 95%CI [-0.145, -0.042]). Similarly, fixation duration was significantly longer on the lower face region (ED-N-back lower ROI: M = 0.335%; 95%CI [0.281, 0.386]). No significant difference was found for the upper face region (ED-N-back upper ROI: M = -0.030%; 95%CI [-0.077, 0.018]; Supplementary Figure 7).

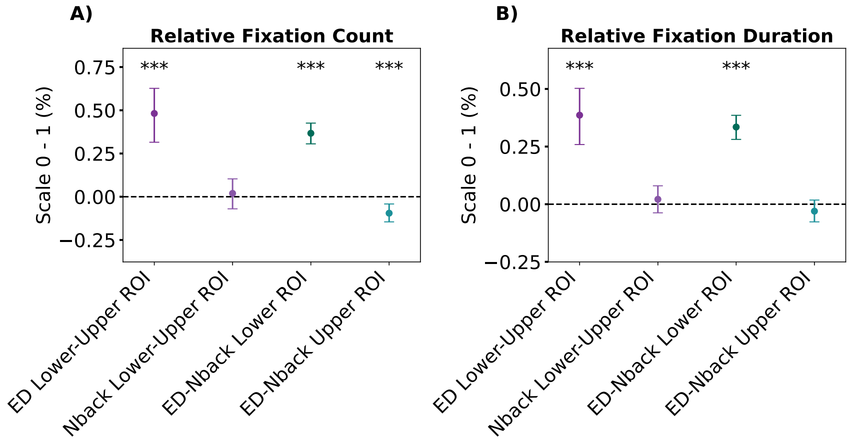

FIGURE 7 Exploratory interaction comparisons of task phase (emotion discrimination, n-back encoding/retrieval) and face region (upper and lower half of the face) for relative fixation count and duration. Coloured dots and error bars represent the bootstrapped grand averages and their Bonferroni-corrected 2.5^{th} and 97.5^{th} confidence interval (CI) across participants. ED: emotion discrimination; Nback: n-back encoding/retrieval phase. Significance level from the linear mixed models: *** for $p < 0.001$, ** for $p < 0.01$, * for $p < 0.05$.

A.3.2 | Face Fixations and Pupil dilation

To compare the two dual-task phases, we extracted pupil dilation as well as the relative fixation count and duration on the face during the emotion discrimination (ED) and n-back encoding/retrieval phase (N-back; full face ROI in Supplementary Figure 6; cf. A.3.1).

In comparing eye behaviour across the two dual-task phases, we found a significant main effect of task phase on relative fixation count ($F_{(1,429)}$ = 1566.05, p < .001) and duration ($F_{(1,429)}$ = 1203.29, p < .001) on the ROI of the face stimulus, as well as pupil dilation during fixations ($F_{(1,429)}$ = 90.24, p < .001). During the emotion discrimination, faces were fixated more frequently (ED – N-back: M = 30.460%; 95%CI [26.485, 34.490]) and for longer durations (ED – N-back: M = 27.188%; 95%CI [23.928, 30.465]), regardless of the displayed emotion or current WM load level. Pupil dilation was increased during emotion discrimination compared to the n-back encoding/retrieval phase (ED – N-back: M = 0.145 mm; 95%CI [0.113, 0.177]).

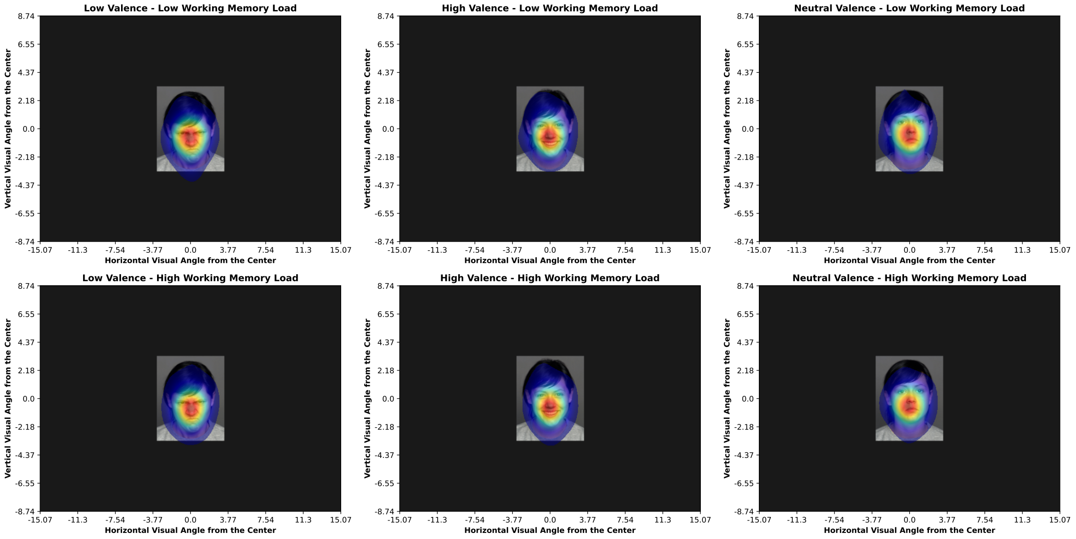

FIGURE 8 Descriptive heatmap of the fixations averaged across subjects per condition during the facial emotion discrimination.

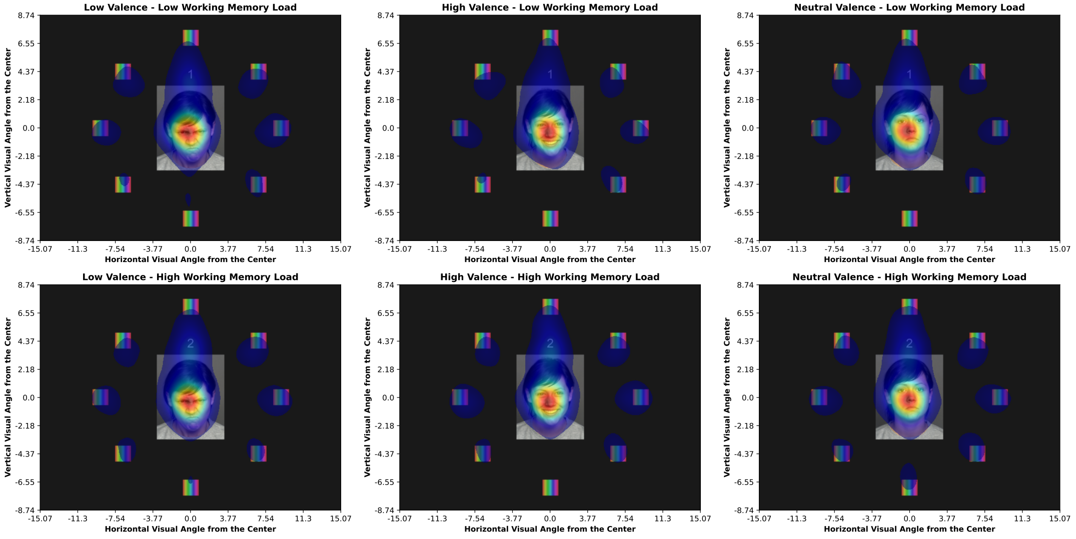

FIGURE 9 Descriptive heatmap of the fixations averaged across subjects per condition during the n-back encoding/retrieval phase.

List of Supplementary Figure Legends

Supplementary Figure 1 Overview of the experiment procedure. The shown order of the conditions is an illustrative example. The provided times are approximated. Conditions were presented randomly within one round without repetition. LV: low valence; NV: neutral valence; HV: high valence; I: instruction.

Supplementary Figure 2 Spatiotemporal F-statistic cluster of the main effect emotional expression and the temporally evolving spatial distribution of modulated event-related magnetic fields (ERFs).

Supplementary Figure 3 Regions of interest (ROIs) comprising the primary peak (blue outline) and secondary peak (magenta outline) per comparison, cluster, and time interval (see Figure 4). A) Caudal perspective for the left hemisphere and B) caudal and inferior (180 - 390 ms) as well as lateral perspective (390 - 1000 ms) for the right hemisphere of the contrast negative - neutral emotional expressions (Figure 4A, B). Lateral perspective for C) the left and D) right hemisphere as well as E) medial (400 - 550 ms), lateral (550 - 700 ms), and frontal (700 - 1000 ms) perspective for the second left hemispheric cluster of the contrast negative - positive emotional expressions (Figure 4C-E). Significant spatiotemporal correlation coefficients within and among the ROIs and time intervals are labelled.

Supplementary Figure 4 Correlation matrices of the trial-by-trial Spearman rank correlations between regions of interest (ROIs). ROIs were extracted from significant spatiotemporal event-related magnetic fields (ERFs) for each comparison, cluster per hemisphere, and time interval of interest (TOI; Figure 4). Labels were defined using the Automated Anatomical Labeling (AAL) atlas and Brodmann atlas. Clu: Cluster; L: left; R: right; LV: low valence; NV: neutral valence; HV: high valence; OFA: occipital face area; FFA: fusiform face area; Mid: middle; Sup: superior; FO: frontal operculum; SMA: supplementary motor area. LV-NV clusters and LV-HV Cluster 1 with TOIs ranging from (1) 190–389 ms, (2) 390–600 ms, (3) 600–800 ms, and (4) 800–1,000 ms. LV-HV Cluster 2 with TOIs ranging from (1) 400–550 ms, (2) 550–700 ms, (3) 700–850 ms, and (4) 850–1,000 ms.

Supplementary Figure 5 Correlation between individual periodic working memory (WM) load-based alpha band modulation (i.e., directional oscillatory changes $HW - LW$) and n-back retrieval reaction time under high WM load. The analysis focuses on individual periodic alpha band power, averaged across cluster vertices identified through a permutation-based cluster analysis. HW: High WM load; LW: Low WM load; r: Spearman rank correlation.

Supplementary Figure 6 Defined regions of interest (ROIs) around the face stimuli (blue circle) and squares (red circles) for the fixation-related eye-tracking analysis.

Supplementary Figure 7 Exploratory interaction comparisons of task phase (emotion discrimination, n-back encoding/retrieval) and face region (upper and lower half of the face) for relative fixation count and duration. Coloured dots and error bars represent the bootstrapped grand averages and their Bonferroni-corrected 2.5^{th} and 97.5^{th} confidence interval (CI) across participants. ED: emotion discrimination; Nback: n-back encoding/retrieval phase. Significance level from the linear mixed models: *** for $p < 0.001$, ** for $p < 0.01$, * for $p < 0.05$.

Supplementary Figure 8 Descriptive heatmap of the fixations averaged across subjects per condition during the facial emotion discrimination.

Supplementary Figure 9 Descriptive heatmap of the fixations averaged across subjects per condition during the n-back encoding/retrieval phase.

References

Bonnefond, M. and Jensen, O. (2012) Alpha oscillations serve to protect working memory maintenance against anticipated distracters. *Current Biology*, **22**, 1969–1974.

Donoghue, T., Haller, M., Peterson, E. J., Varma, P., Sebastian, P., Gao, R., Noto, T., Lara, A. H., Wallis, J. D., Knight, R. T.,

Shestyuk, A. and Voytek, B. (2020) Parameterizing neural power spectra into periodic and aperiodic components. *Nature Neuroscience*, **23**, 1655–1665.

Gramfort, A., Luessi, M., Larson, E., Engemann, D. A., Strohmeier, D., Brodbeck, C., Goj, R., Jas, M., Brooks, T., Parkkonen, L. and Hämäläinen, M. (2013) Meg and eeg data analysis with mne-python. *Frontiers in Neuroscience*, **7**, 267.

Gyurkovics, M., Clements, G. M., Low, K. A., Fabiani, M. and Gratton, G. (2021) The impact of 1/f activity and baseline correction on the results and interpretation of time-frequency analyses of eeg/meg data: A cautionary tale. *Neuroimage*, **237**, 118192.

Lin, F.-H., Witzel, T., Ahlfors, S. P., Stufflebeam, S. M., Belliveau, J. W. and Hämäläinen, M. S. (2006) Assessing and improving the spatial accuracy in meg source localization by depth-weighted minimum-norm estimates. *NeuroImage*, **31**, 160–171.

Proskovec, A. L., Wiesman, A. I., Heinrichs-Graham, E. and Wilson, T. W. (2019) Load effects on spatial working memory performance are linked to distributed alpha and beta oscillations. *Human Brain Mapping*, **40**, 3682–3689.
